# Supplementary material for: Towards Protection of Nucleic Acids from Herbicide Attack: Self-Assembly of Betaines Based on Pillar[5]arene with Glyphosate and DNA
Source: Int J Mol Sci. 2023 May 6;24(9):8357. doi: 10.3390/ijms24098357 (PMC10179701; doi:10.3390/ijms24098357)
Supplement: Supplementary file 1 [file ijms-24-08357-s001.zip › ijms-2357581-supplementary.pdf]

Towards protection of nucleic acids from herbicide attack: self-assembly of betaines based on pillar[5]arene with glyphosate and DNA

Anastasia Nazarova[1,*], Pavel Padnya[1], Arthur Khannanov[1], Aleksandra Khabibrakhmanova[2], Pavel Zelenikhin[2], Ivan Stoikov[1,3,*]

[1] A.M. Butlerov Chemistry Institute of Kazan Federal University, 18 Kremlyovskaya Str., 420008 Kazan, Russia; padnya.ksu@gmail.com

[2] Institute of Fundamental Medicine and Biology of Kazan Federal University, 18 Kremlyovskaya Str., 420008 Kazan, Russia; pasha_mic@mail.ru (P.Z.); sasha_xb@mail.ru (A.Kh.)

[3] Federal Center for Toxicological, Radiation and Biological Safety, 2 Nauchny Gorodok Str., Kazan 420075, Russia

*Correspondence: anas7tasia@gmail.com (A.N.); ivan.stoikov@mail.ru (I.S.)

Electronic Supplementary Information (31 pages)

Table of Contents

1. Materials and Methods S3
2. ^{1}H NMR spectra S5
3. ^{13}C NMR spectra S7
4. ESI mass-spectra S9
5. FTIR spectra S11
6. ^{1}H-^{1}H NOESY NMR spectra S13
7. DLS data S13–S18, S29
8. Bindfit data S19, S22, S23, S25-S28
9. Electron absorption spectra S20, S21, S24
10. Cytotoxicity S30
11. References S31

1. Materials and Methods

1.1. General

^{1}H NMR, ^{13}C and 2D NOESY NMR spectra were obtained on a Bruker Avance-400 spectrometer (Bruker Corp., Billerica, MA, USA) (^{13}C{1H} – 100 MHz and ^{1}H and 2D NOESY – 400 MHz). The chemical shifts were determined against the signals of residual protons of deuterated solvent (DMSO-d_6). The concentrations of the compounds were equal to 3–5% by the weight in all the records. The FTIR ATR spectra were recorded on the Spectrum 400 FT-IR spectrometer (Perkin Elmer Inc, Waltham, MA, USA) with a Diamond KRS-5 attenuated total internal reflectance attachment (resolution 0.5 cm^{-1}, accumulation of 64 scans, recording time 16 s in the wavelength range 400–4000 cm^{-1}). Mass spectra (ESI) were recorded on an AmaZonX mass spectrometer (Bruker Daltonik GmbH, Bremen, Germany). The drying gas was nitrogen at 300 °C. The capillary voltage was 4.5 kV. The samples were dissolved in acetonitrile (concentration ~ 10^{-6} g/mL). ESI HRMS experiments were performed at Agilent 6550 iFunnel Q-TOF LC/MS (Agilent Technologies, Santa Clara, CA, USA), equipped with Agilent 1290 Infinity II LC. Melting points were determined using Boetius Block apparatus (VEB Kombinat Nagema, Radebeul, Germany). Chemically pure organic solvents were purified by standard methods. All the aqueous solutions were prepared with the Millipore-Q deionized water (>18.0 MW cm at 298 K).
Pillar[5]arene **1** and **2** was synthesized according to the literature [1].

1.2. Dynamic Light Scattering (DLS)

1.2.1. Particles' Size

The distribution of particles by number, volume, and intensity, the polydispersity index were determined by dynamic light scattering (DLS) on a Zetasizer Nano ZS instrument (Malvern Instruments, Worcestershire, UK) in quartz cuvettes. The instrument is equipped with the 4 mW He-Ne laser (633 nm). Measurements were performed at a detection angle of 173°. The error in determining the particle size is less than 2%. The results were processed by the DTS program (Dispersion Technology Software 4.20). Deionized water was used to prepare solutions for studying the aggregation of pillar[5]arenes **1**-**4**. In the course of the experiment, the concentrations of macrocycles varied within 1 × 10^{-6} – 1 × 10^{-4} M. 10 mM Tris-HCl buffer (pH = 7.4) was used as a solvent to determine the size of the triple systems (pillar[5]arene/DNA/GIS). Triple systems (pillar[5]arene-DNA-GIS) were prepared similarly to those studied by the UV-Vis spectroscopy. The particle sizes were measured after 1 h mixing. Measurements were determined after 24 and 178 h three times to evaluate kinetic stability.

1.3. Electrophoretic Light Scattering (ELS)

1.3.1. Zeta Potentials

Zeta (ζ) potentials were determined by electrophoretic light scattering (ELS) on a Zetasizer Nano ZS from Malvern Instruments (Worcestershire, UK). Samples were prepared as for the DLS measurements and were transferred with the syringe to the disposable folded capillary cell for measurement. The zeta potentials were measured using the Malvern M3-PALS method and averaged from three measurements.

1.4. Transmission electron microscopy (TEM)

TEM measurements were made at the Interdisciplinary Center for Analytical Microscopy of the Kazan Federal University. Analysis of samples was carried out using a Hitachi HT7700 Exalens transmission electron microscope (Tokyo, Japan) with an Oxford Instruments X-Maxn 80T EDS detector working in STEM mode. Samples of pillar[5]arene **2** (1 × 10^{-4} M), and triple system pillar[5]arene **2**-DNA-GIS were prepared similarly to those studied by the DLS method. Deionized water was used as a solvent. 10 μl of the suspension was placed on a carbon-coated 3 mm copper grid and dried at room temperature using special holder for microanalysis. After drying, the grid was placed in the transmission electron microscope and analyzed at an accelerating voltage of 80 kV.

1.5. UV-Spectroscopy

Absorption spectra were recorded on a Shimadzu UV-3600 spectrometer (Kyoto, Japan). Quartz cuvettes with an optical path length of 10 mm were used. Tris-HCl buffer was used for preparation of the solutions. Salmon sperm DNA (Sigma) was used as received. The purity of the DNA was checked by the ratio of the absorbance A260/A280 > 1.8, indicating the DNA was sufficiently free from protein. Concentration of DNA was 2.5×10^{-7} M. Concentrations of GIS and pillar[5]arenes **1**-**4** were 3×10^{-6} M. Absorption spectra of mixtures were recorded after an 1 h incubation at 20 °C.

1.5.1. Determination of the Stability Constant and Stoichiometry of the Complex by Spectrophotometric Titration

3 × 10^{-5} M solution of GIS (9, 15, 18, 30, 45, 60, 90, 150, 210 and 300 µL) in Tris-HCl buffer was added to 25 µL of a solution of DNA (1 × 10^{-5} M) in Tris-HCl buffer and diluted to final volume of 3 mL with Tris-HCl buffer solution. The UV spectra of the solutions were then recorded. The stability constant of complex was calculated by Bindfit. Three independent experiments were carried out for each series.

3 × 10^{-5} M solution of pillar[5]arene **1** (**4**) (30, 60, 90, 120, 150, 180, 210, 240, 270 and 300 µL) in Tris-HCl buffer was added to 25 µL of a solution of DNA (1 × 10^{-5} M) in Tris-HCl buffer and diluted to final volume of 3 mL with Tris-HCl buffer solution. The UV spectra of the solutions were then recorded. The stability constant of each complex was calculated by Bindfit. Three independent experiments were carried out for each series.

25 µL of a solution of DNA (1 × 10^{-5} M) in Tris-HCl buffer was added to 300 µL of a solution of macrocycle (**1**-**4**) with a concentration of 3×10^{-5} M, after which 3 × 10^{-5} M solution of GIS (9, 15, 18, 30, 45, 60, 90, 150, 210 and 300 µL) in Tris-HCl buffer was added to the resulting associate and diluted to final volume of 3 mL with Tris-HCl buffer solution. The UV spectra of the solutions were then recorded. The stability constant of complex was calculated by Bindfit. Three independent experiments were carried out for each series.

*1.6. Cytotoxicity of pillar[5]arenes **1**–**4**, GIS and their mixtures on A549 and LEC Cell Lines*

The ability of macrocyclic compounds **1**–**4**, GIS and their mixtures to inhibit the viability and proliferative activity of LEC cells and A549 cells was investigated using the MTT test according to [2]. A549 – human alveolar adenocarcinoma cell line (ATCC, Rockville, Maryland, USA) and cow embryo lung epithelial (LEC) cells (Animal Cell Culture Collection of Russian Academy of Agricultural Science, Kazan, Russia) were cultured in DMEM (GIBCO, Waltham, MA, USA) medium supplemented with 10% serum and 100 units/mL of penicillin (PanEco, Moscow, Russia) and streptomycin (PanEco, Moscow, Russia) in a humidified atmosphere with 5% CO_2 at 37 °C. Cells were seeded in 96-well plates at a concentration of 10^4 cells/well. After 24 h of cultivation, the medium was removed from the wells and replaced with a fresh one with the addition of the test substances. The volume of the culture medium in the wells was 100 µL. After 24 h incubation of cells in the presence of substances, the medium in the wells was replaced with a fresh one containing MTT reagent at a final concentration of 0.5 mg/mL. The cells were incubated with MTT for 4 h at 37 °C in an atmosphere of 5% CO_2. Then, the medium was aspirated from the wells and 100 µL of dimethyl sulfoxide was added. Probes were incubated at 37 °C for 15 min in the dark for the formazan crystals to dissolve. The optical density of the formazan solution in the wells was measured using a plate reader (BioRad xMarkTM Microplate Spectrophotometer, USA) at a wavelength of 570 nm. Three series of experiments were carried out with at least 8 replications for each variant in the series.

*1.7. Characterization of pillar[5]arene **2** Penetration into A549 and LEC Cells by Flow Cytometry*

The macrocyclic compounds' ability to induce apoptosos of *A549* and *LEC* cells was determined with a BD FACSCanto II flow cytometer. *A549* and *LEC* cells were grown in DMEM (GIBCO, Waltham, MA, USA) after supplementing with 10% FBS (Corning, Inc., Corning, NY, USA), 100 units/mL penicillin (PanEco, Moscow, Russia) and 100 µg/mL streptomycin (PanEco, Moscow, Russia), at 37 °C in a humidified atmosphere with 5% CO_2. Cells were seeded in 6-well plates at a concentration of 10^6 cells/well and were incubated overnight to attach in a humidified atmosphere with 5% CO_2 at 37 °C. Then the medium was removed from the wells and replaced with a fresh one with the addition of the test substances. The volume of the culture medium in the wells was 2 mL. After 24 h of incubation cells were trypsinised, replaced into centrifuge tubes and centrifuged at 2000 rpm for 5 min at room temperature. Then the cell pellet was resuspended with complete medium and stained with 20 µ L of 3,3′-dihexyloxacarbocyanine iodide ($DiOC_6$) solution (10 µM) in the dark at room temperature for 30 min [3]. Then the cell suspension was centrifuged at 2000 rpm for 5 min at room temperature, and cells were resuspended in 1 mL phosphate-buffered saline (PBS, PanEco, Moscow, Russia). The cells were transferred to cytometric tubes, when the samples were stained with 5 µL of PI solution (5 mg/mL) [3], kept in the dark at room temperature for 2 min, and cytometric analysis was performed. The processing of cytometric data was carried out in the FACSDiva application.

Fig. S1. [1]**H NMR spectrum of 4,8,14,18,23,26,28,31,32,35-decakis-[(*N*-[3'-(dimethyl{[(oxidocarbonylmethyl)aminocarbonylmethyl]aminocarbonylmethyl}ammonio)propyl]aminocarbonylmethoxy]-pillar[5]arene (3), DMSO-*d*₆, 298 K, 400 MHz.**

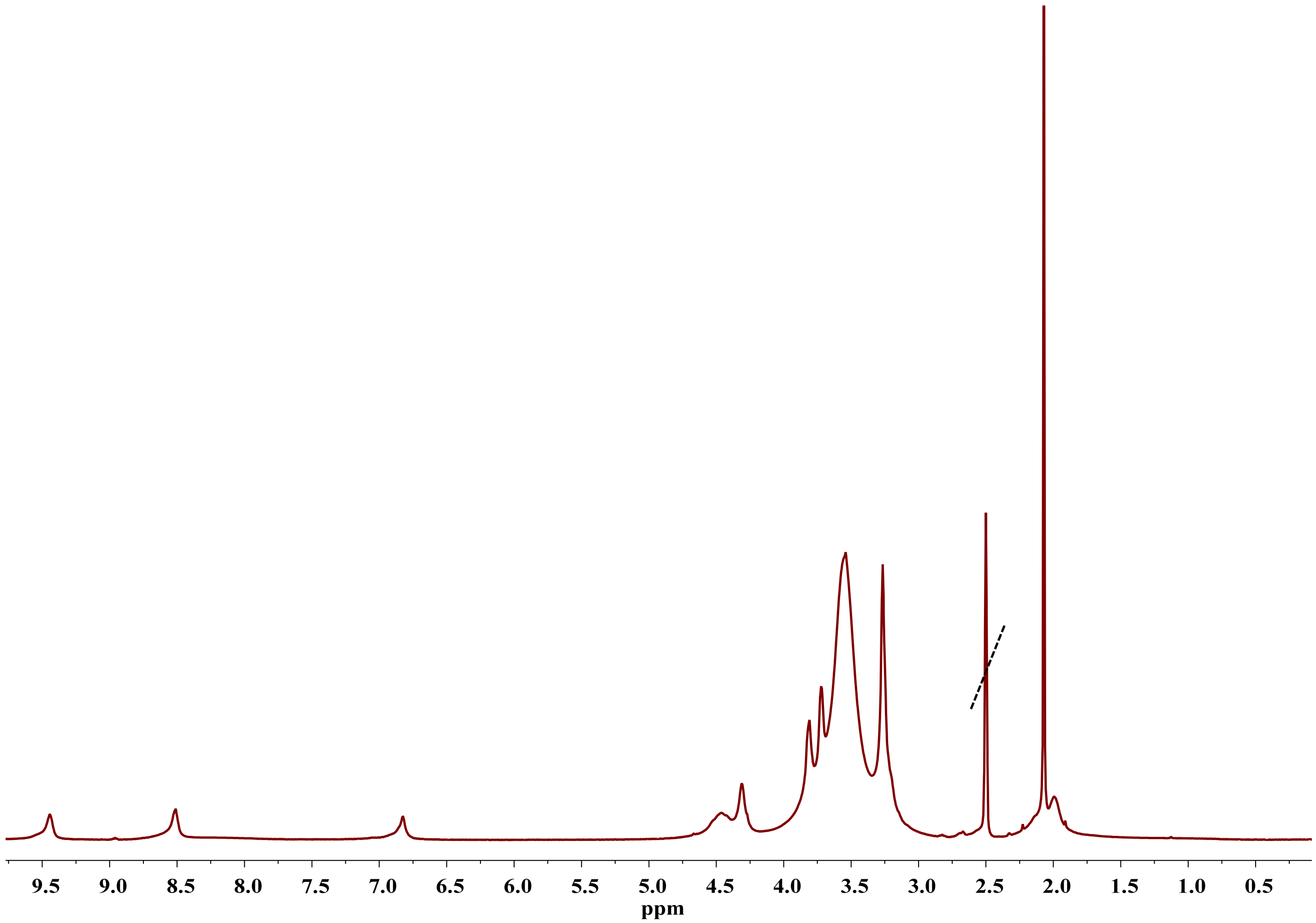

Fig. S2. **^{1}H NMR spectrum of 4,8,14,18,23,26,28,31,32,35-decakis-[(*N*-[3-dimethyl({oxidocarbonyl[*S*-benzyl]methyl}aminocarbonylmethyl)ammonio]propyl)aminocarbonylmethoxy]-pillar[5]arene (4), DMSO-d_6, 298 K, 400 MHz.**

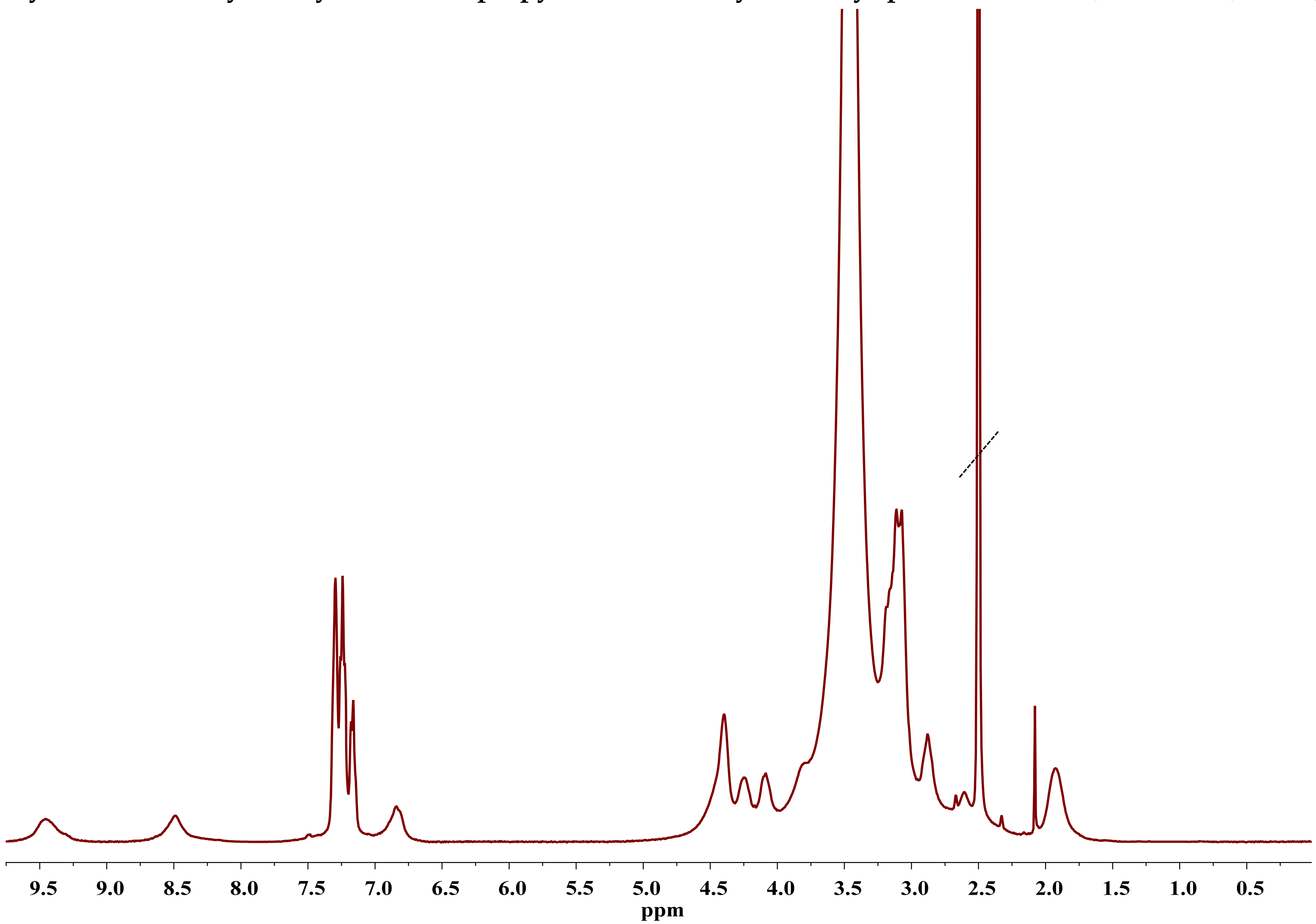

Fig. S3. ^{13}C NMR spectrum of 4,8,14,18,23,26,28,31,32,35-decakis-[(*N*-[3'-(dimethyl{[(oxidocarbonylmethyl)aminocarbonylmethyl]aminocarbonylmethyl}ammonio)propyl]aminocarbonylmethoxy]-pillar[5]arene (3), DMSO-d_6, 298 K, 100 MHz.

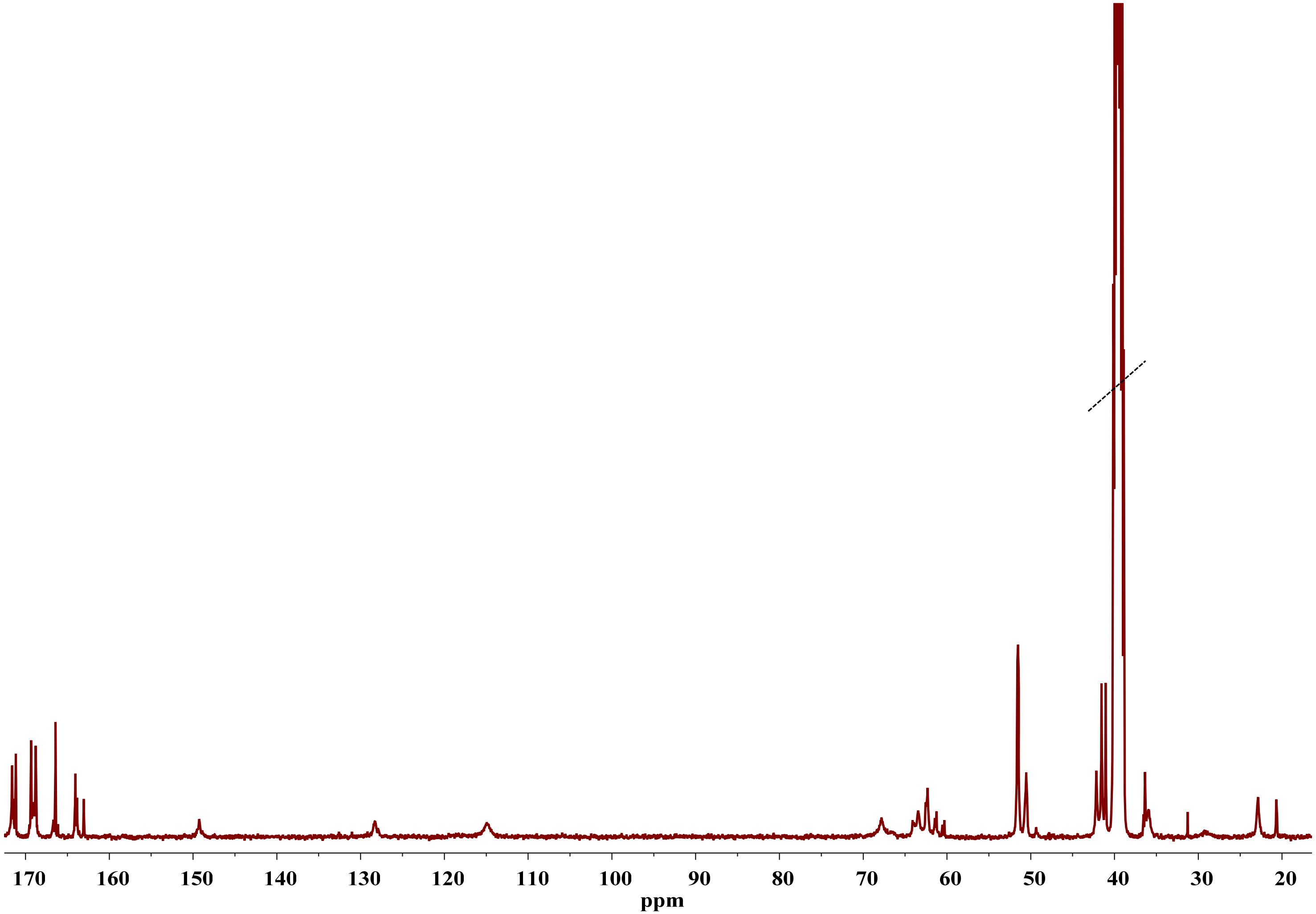

Fig. S4. ^{13}C **NMR spectrum of 4,8,14,18,23,26,28,31,32,35-decakis-[(*N*-[3-dimethyl({oxidocarbonyl[*S*-benzyl]methyl}aminocarbonylmethyl)ammonio]propyl)aminocarbonylmethoxy]-pillar[5]arene (4), DMSO-*d*$_6$, 298 K, 100 MHz.**

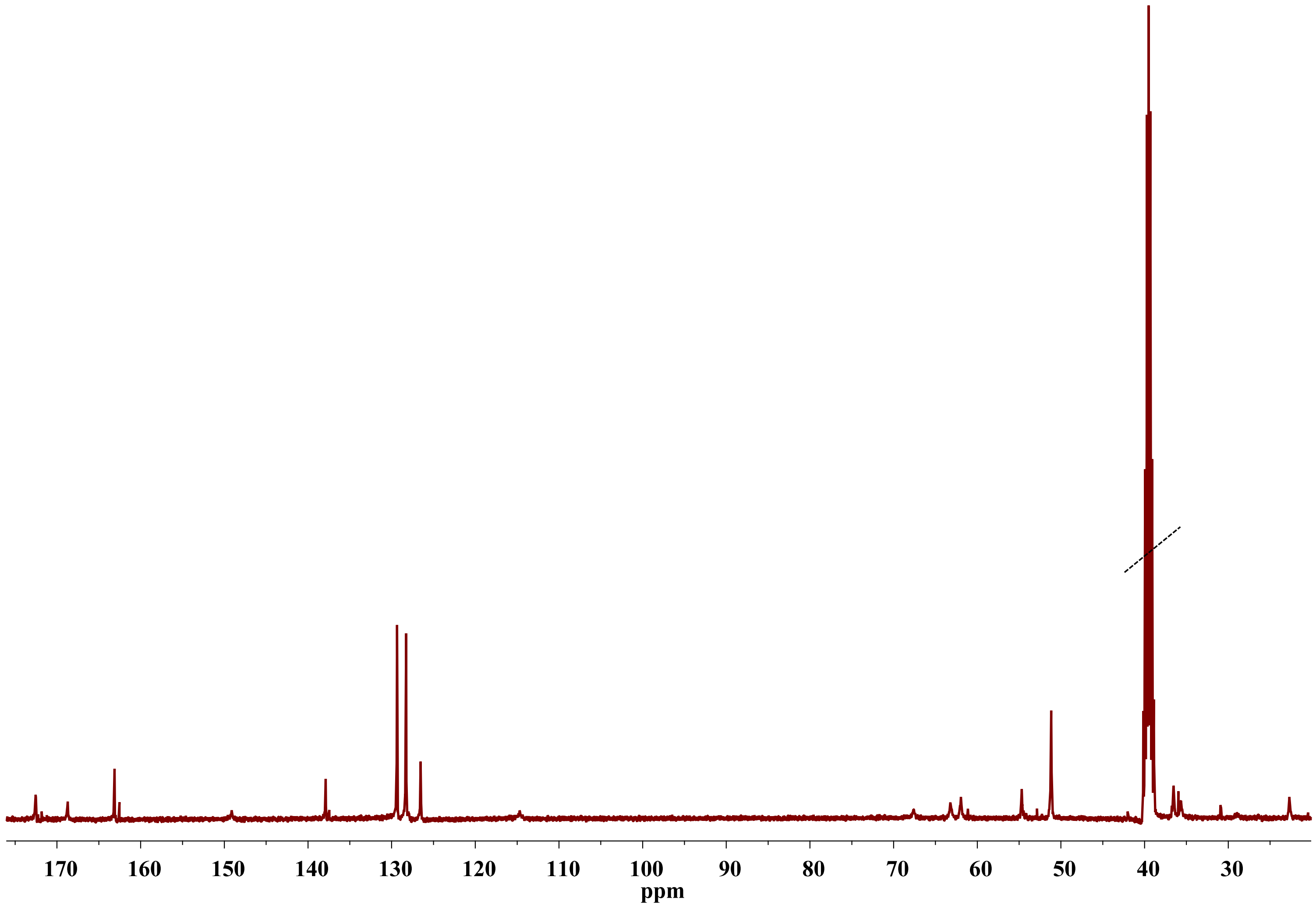

Fig. S5. Mass spectrum (ESI) of 4,8,14,18,23,26,28,31,32,35-decakis-[(*N*-[3'-(dimethyl{[(oxidocarbonylmethyl)aminocarbonylmethyl]aminocarbonylmethyl}ammonio)propyl]aminocarbonylmethoxy]-pillar[5]arene (3).

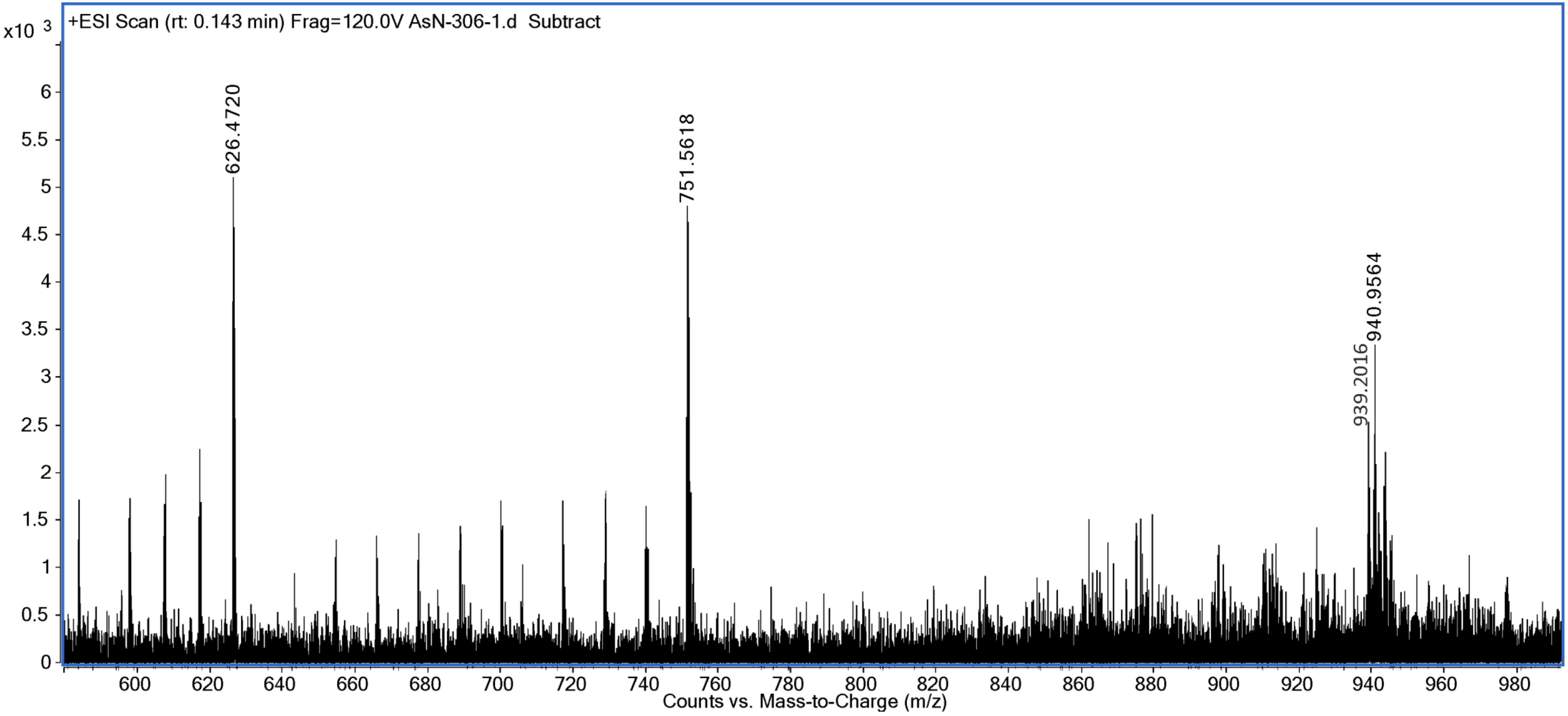

Fig. S6. Mass spectrum (ESI) of 4,8,14,18,23,26,28,31,32,35-Decakis-[(*N*-[3-dimethyl({oxidocarbonyl[*S*-benzyl]methyl}aminocarbonylmethyl)ammonio]propyl)aminocarbonylmethoxy]-pillar[5]arene (4).

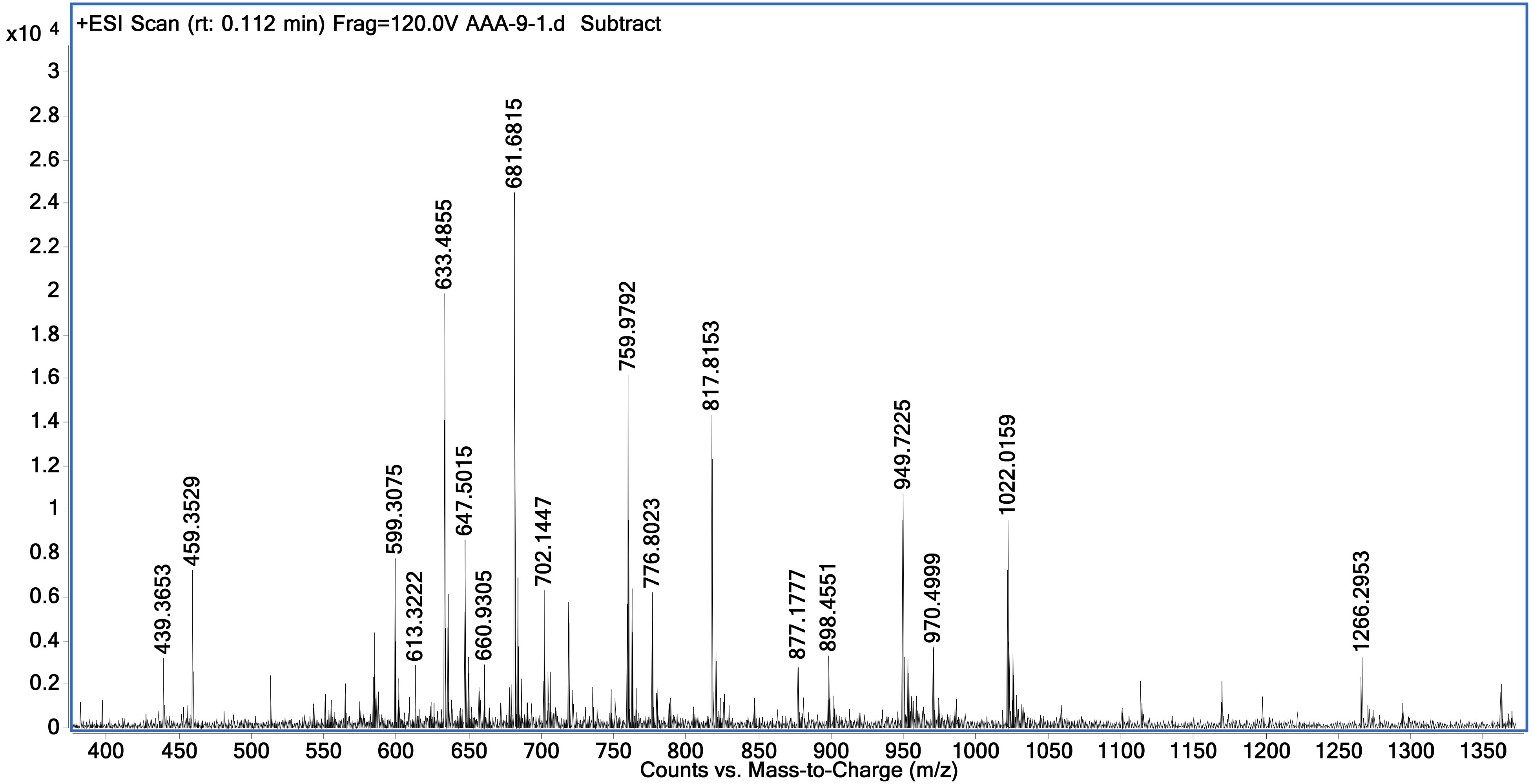

Fig. S7. FTIR spectrum of 4,8,14,18,23,26,28,31,32,35-decakis-[(*N*-[3'-(dimethyl{[(oxidocarbonylmethyl)aminocarbonylmethyl]aminocarbonylmethyl}ammonio)propyl]aminocarbonylmethoxy]-pillar[5]arene (3).

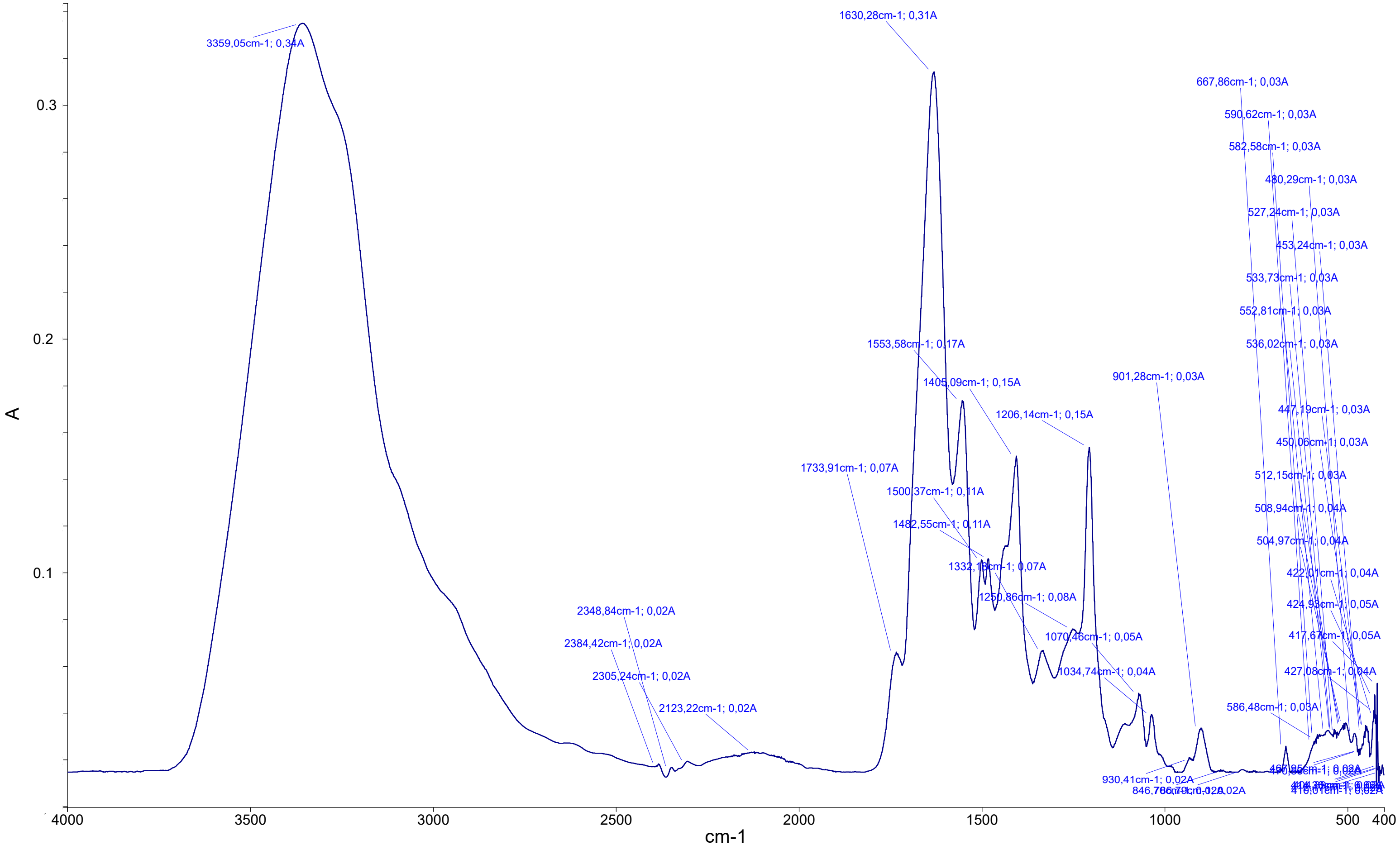

Fig. S8. FTIR spectrum of 4,8,14,18,23,26,28,31,32,35-decakis-[(*N*-[3-dimethyl({oxidocarbonyl[*S*-benzyl]methyl}aminocarbonylmethyl)ammonio]propyl)aminocarbonylmethoxy]-pillar[5]arene (4).

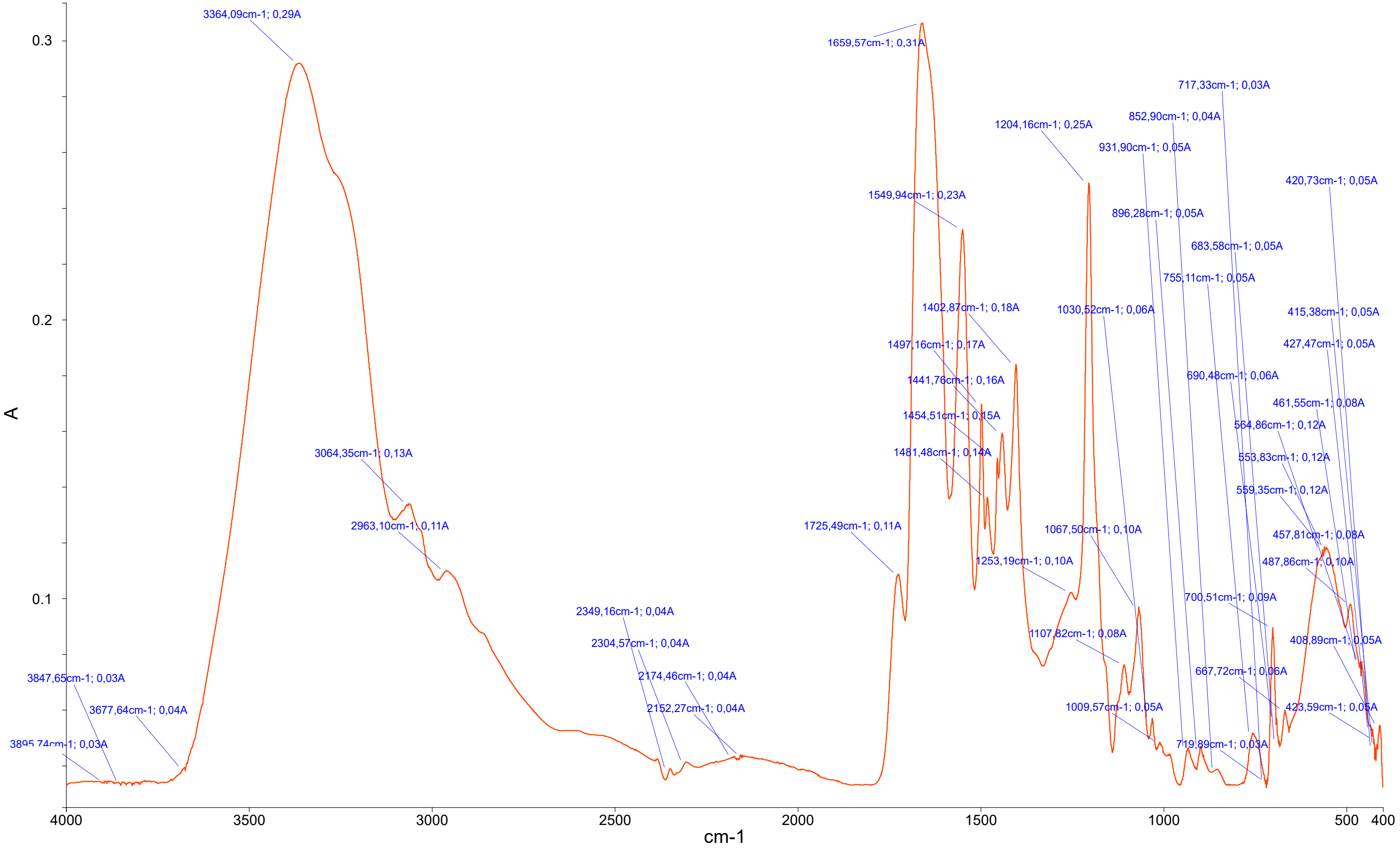

Fig. S9. (a) ^{1}H-^{1}H NOESY NMR spectrum of pillar[5]arene 2 (DMSO-d_6, 298 K, 400 MHz); (b) Proposed intramolecular interactions for pillar[5]arene 2.

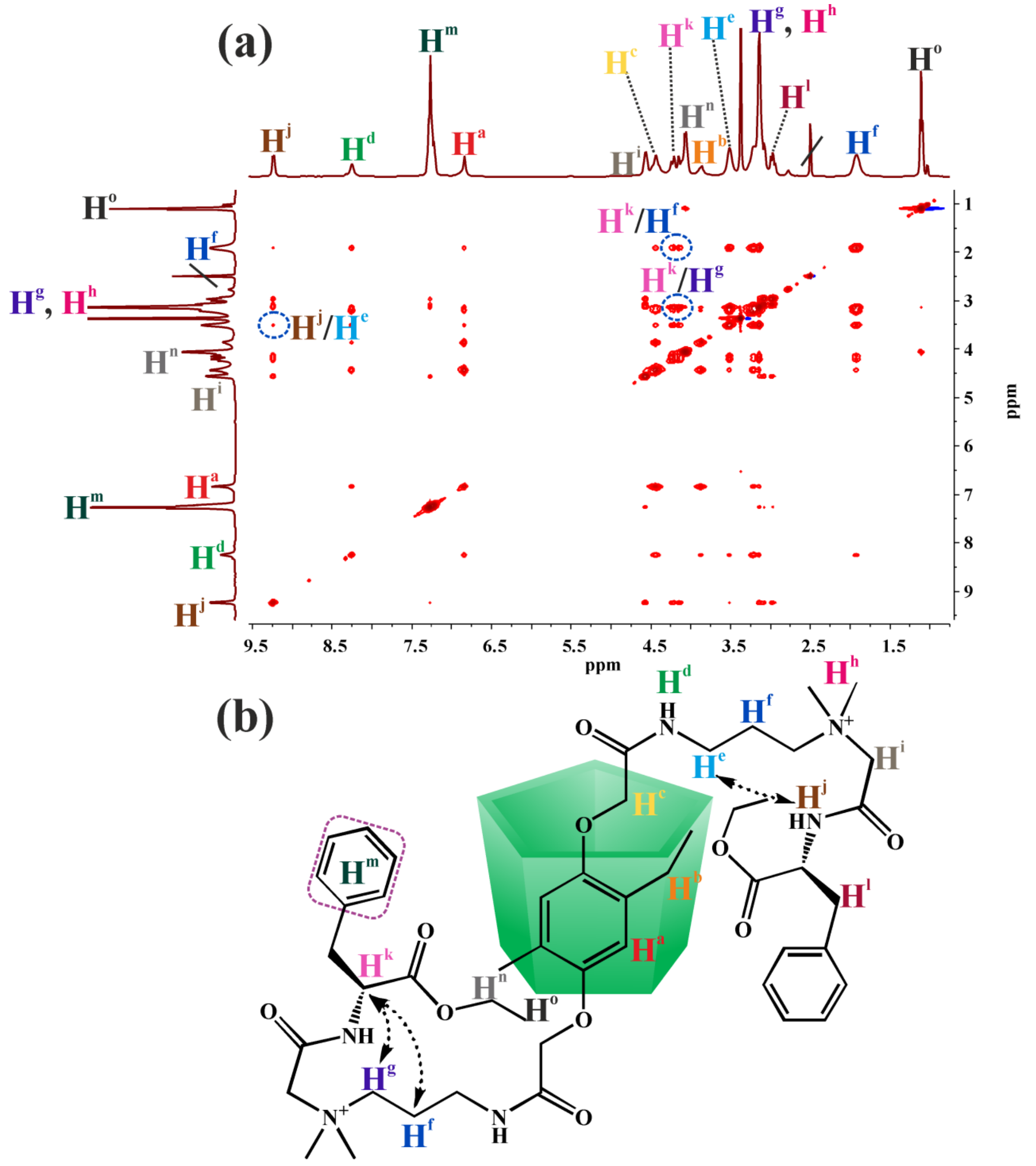

Fig. S10. (a) ^{1}H-^{1}H NOESY NMR spectrum of pillar[5]arene 3 (DMSO-d_6, 298 K, 400 MHz); (b) proposed intramolecular interactions for pillar[5]arene 3; (c) proposed intramolecular interactions for pillar[5]arene 1; (d) ^{1}H-^{1}H NOESY NMR spectrum of pillar[5]arene 1 (DMSO-d_6, 298 K, 400 MHz).

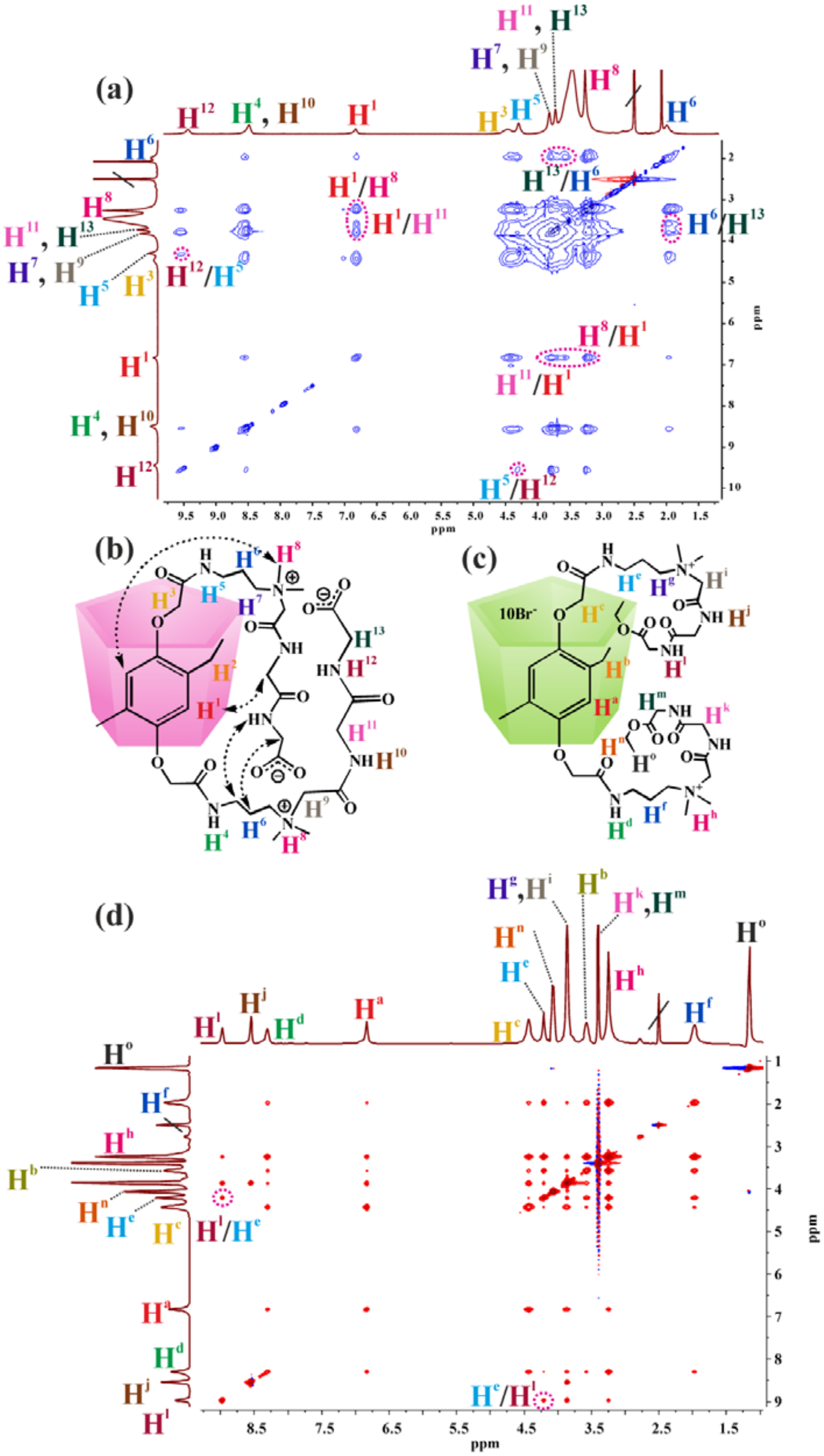

Fig. S11. Size distribution of particles by intensity for macrocycle 1 (1×10^{-6} M) in H_2O.

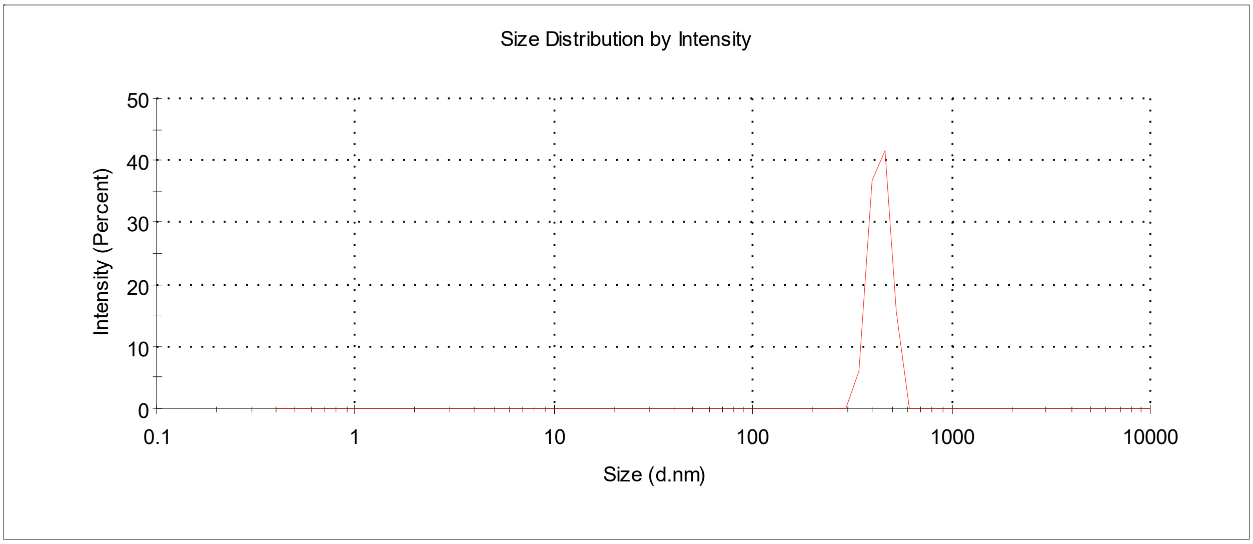

Fig. S12. Size distribution of particles by intensity for macrocycle 1 (1×10^{-5} M) in H_2O.

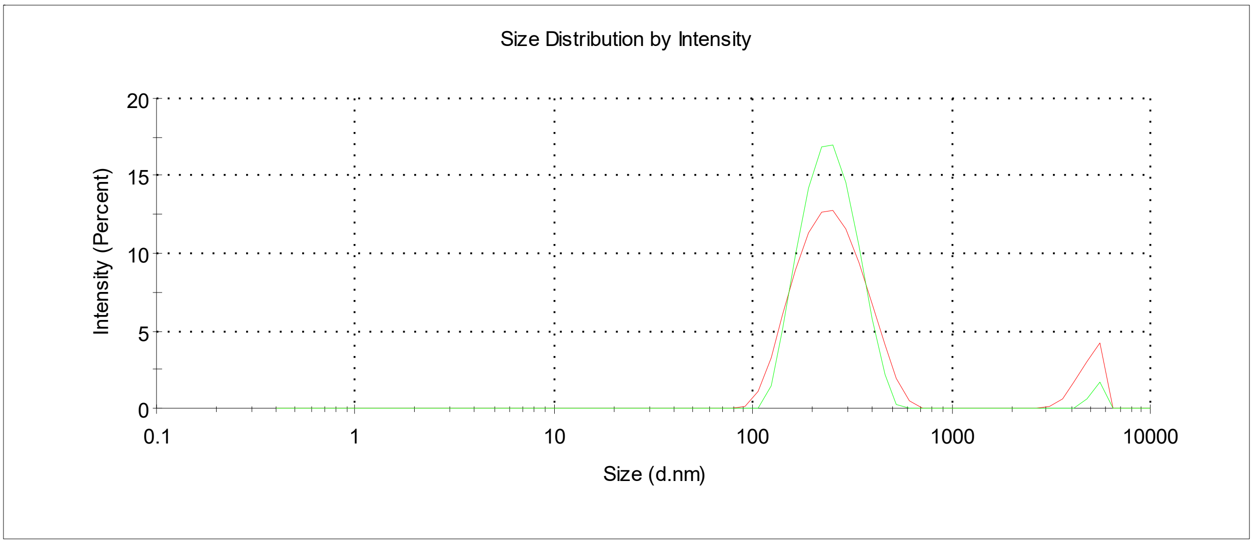

Fig. S13. Size distribution of particles by intensity for macrocycle 1 (1×10^{-4} M) in H_2O.

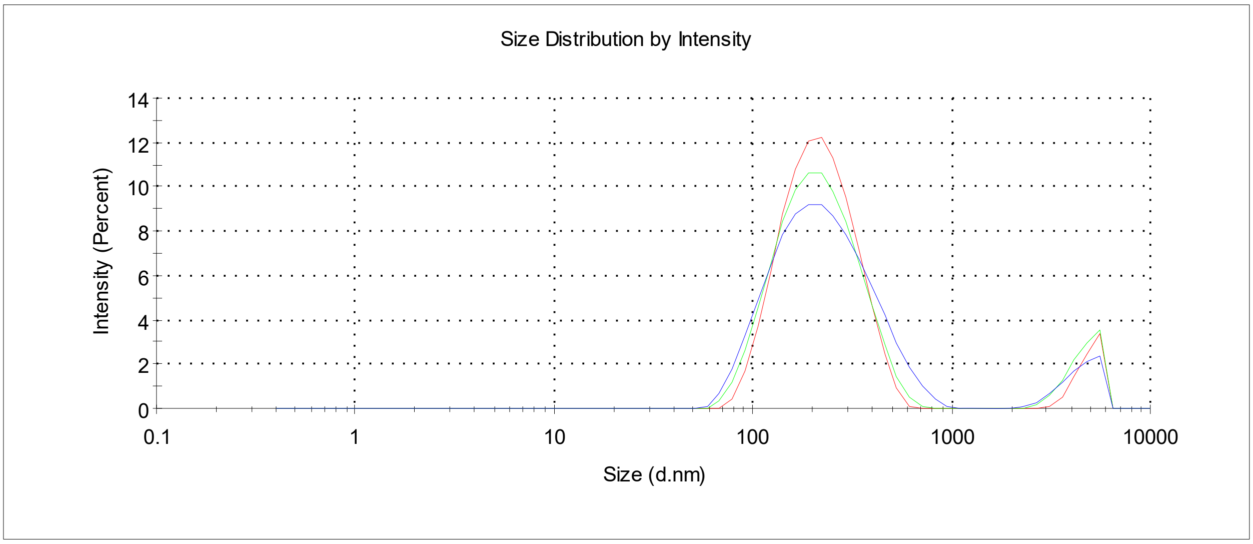

Fig. S14. Size distribution of particles by intensity for macrocycle 2 (1×10^{-6} M) in H_2O.

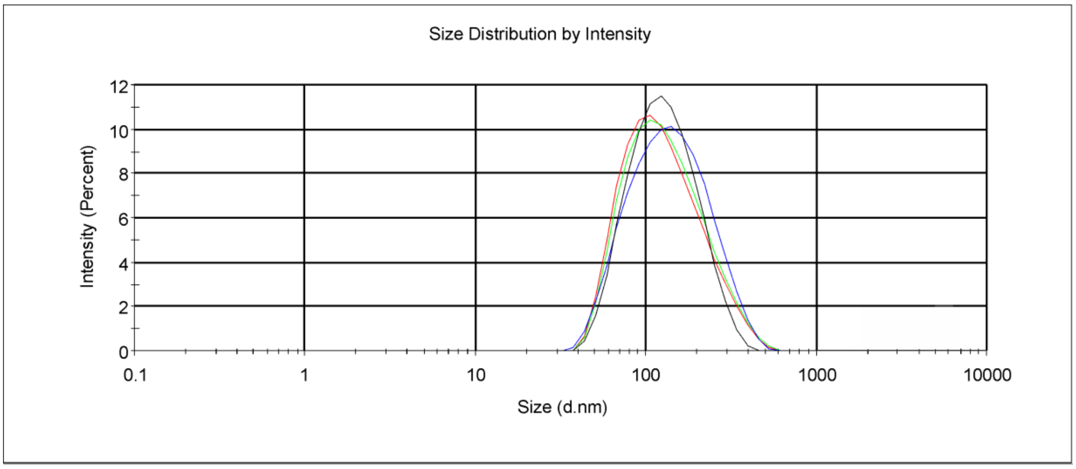

Fig. S15. Size distribution of particles by intensity for macrocycle 2 (1×10^{-5} M) in H_2O.

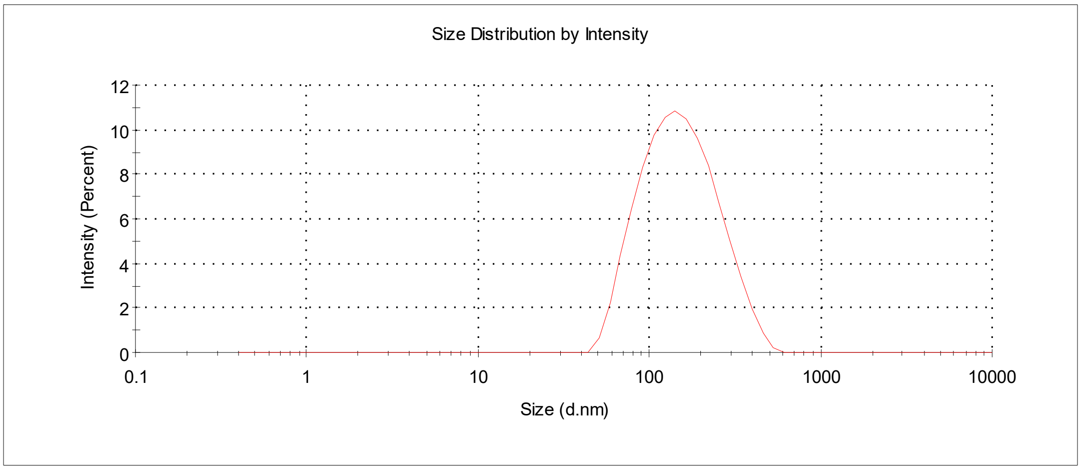

Fig. S16. Size distribution of particles by intensity for macrocycle 2 (1×10^{-4} M) in H_2O.

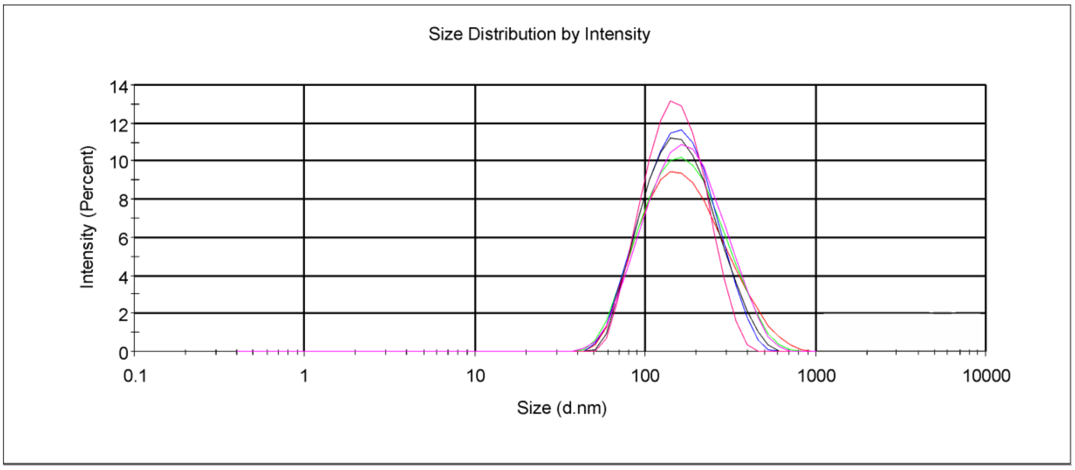

Fig. S17. Size distribution of particles by intensity for macrocycle 3 (1×10^{-6} M) in H_2O.

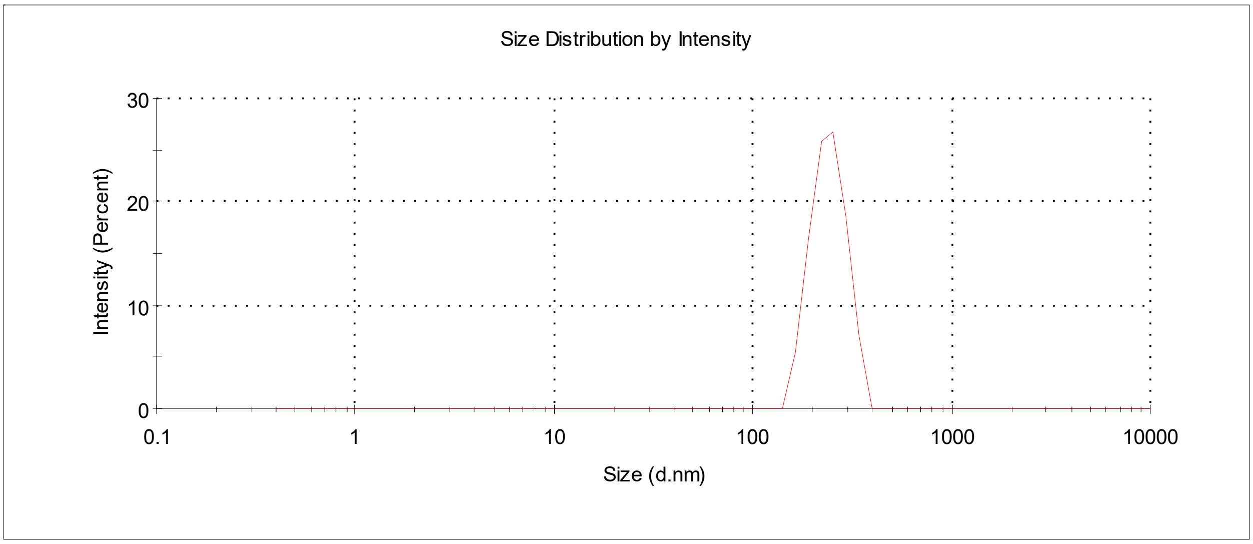

Fig. S18. Size distribution of particles by intensity for macrocycle 3 (1×10^{-5} M) in H_2O.

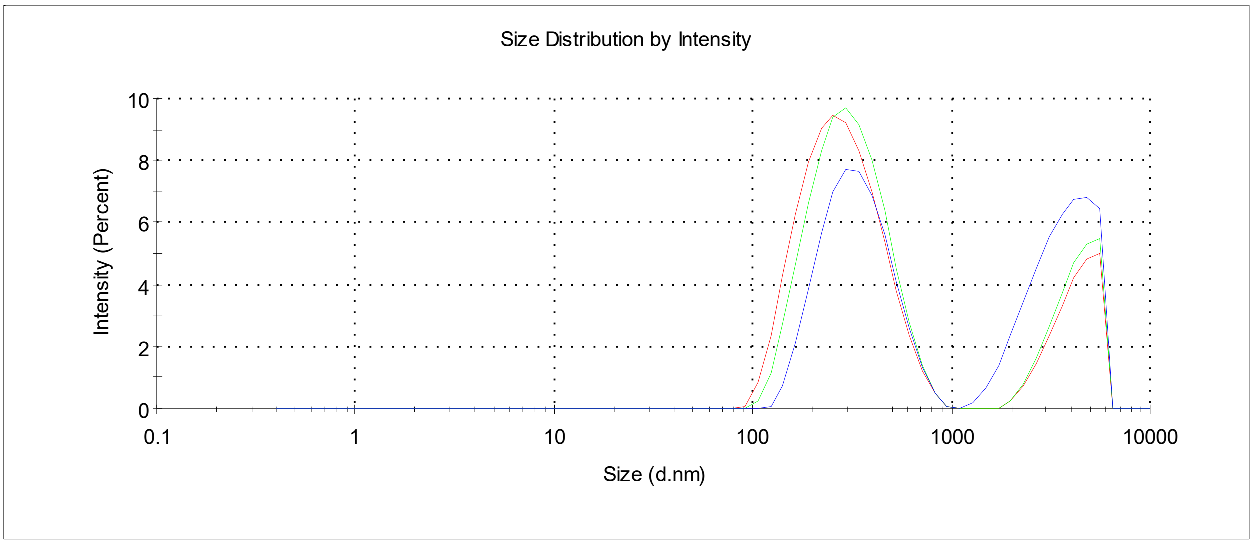

Fig. S19. Size distribution of particles by intensity for macrocycle 3 (1×10^{-4} M) in H_2O.

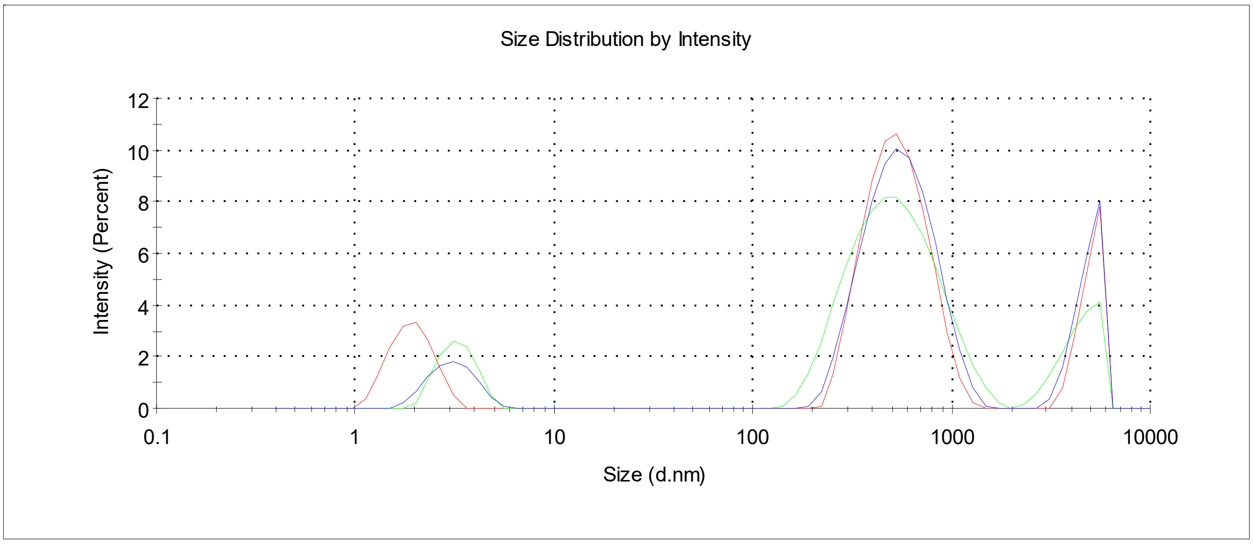

Fig. S20. Size distribution of particles by intensity for macrocycle 4 (1×10^{-6} M) in H_2O.

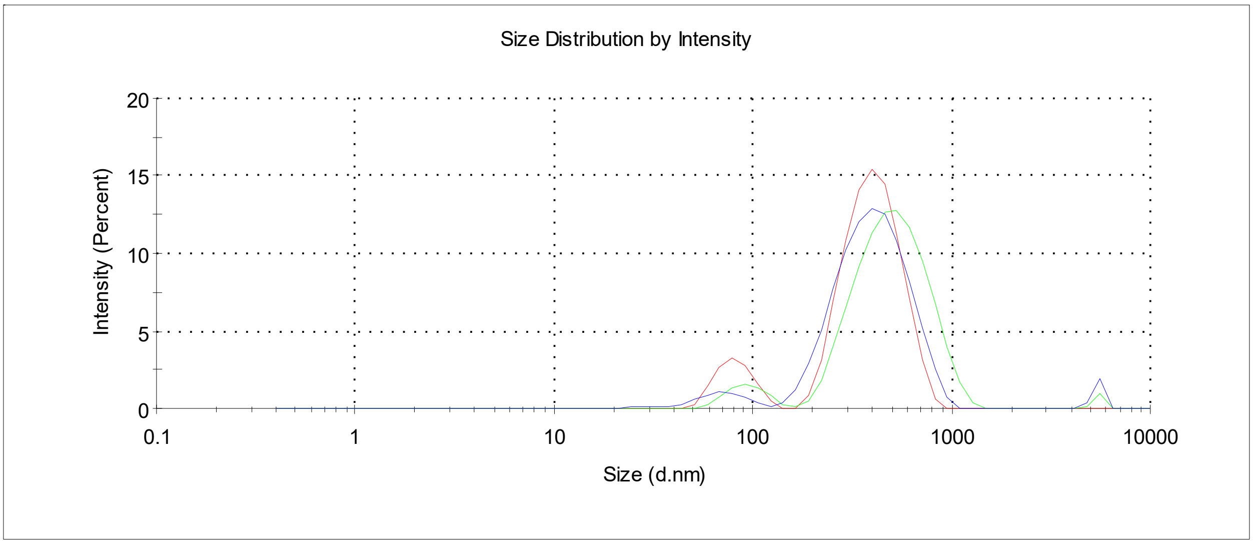

Fig. S21. Size distribution of particles by intensity for macrocycle 4 (1×10^{-5} M) in H_2O.

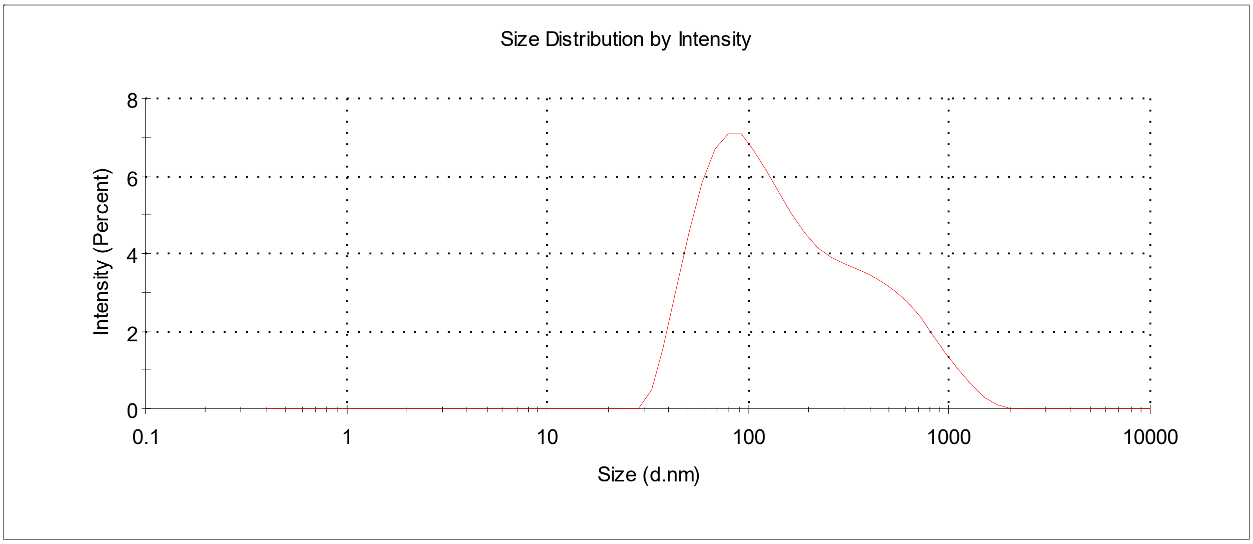

Fig. S22. Size distribution of particles by intensity for macrocycle 4 (1×10^{-4} M) in H_2O.

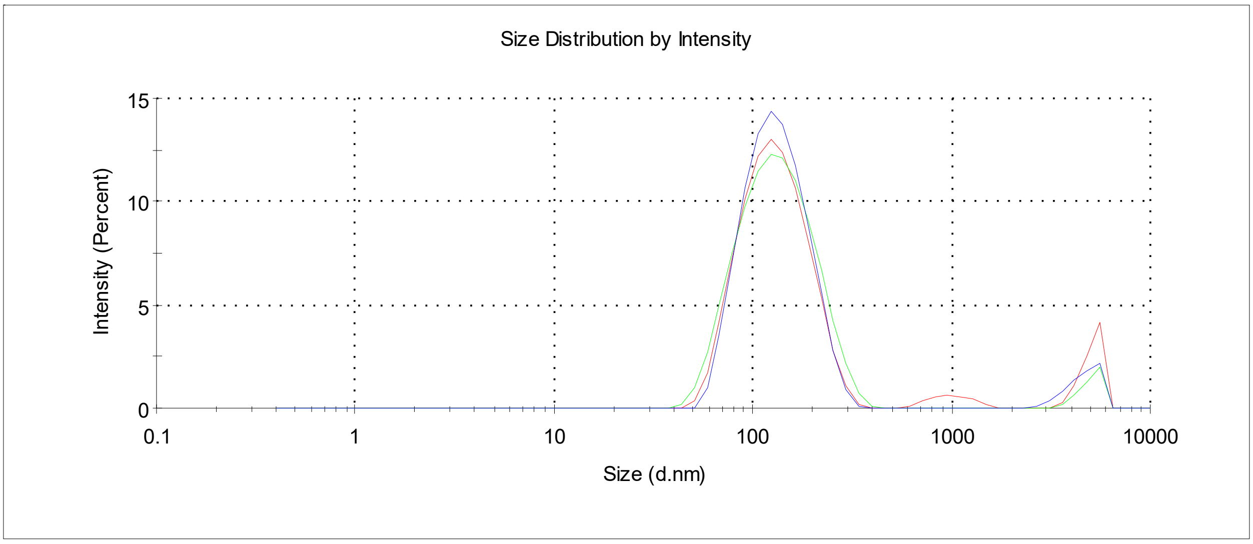

Fig. S23. Bindfit (Fit data to 1:1, 1:2 and 2:1 Host-Guest equilibria) Screenshots taken from the summary window of the website supramolecular.org. This screenshots shows the raw data for UV-vis titration of DNA with GIS in Tris-HCl buffer, the data fitted to 1:1 binding model.

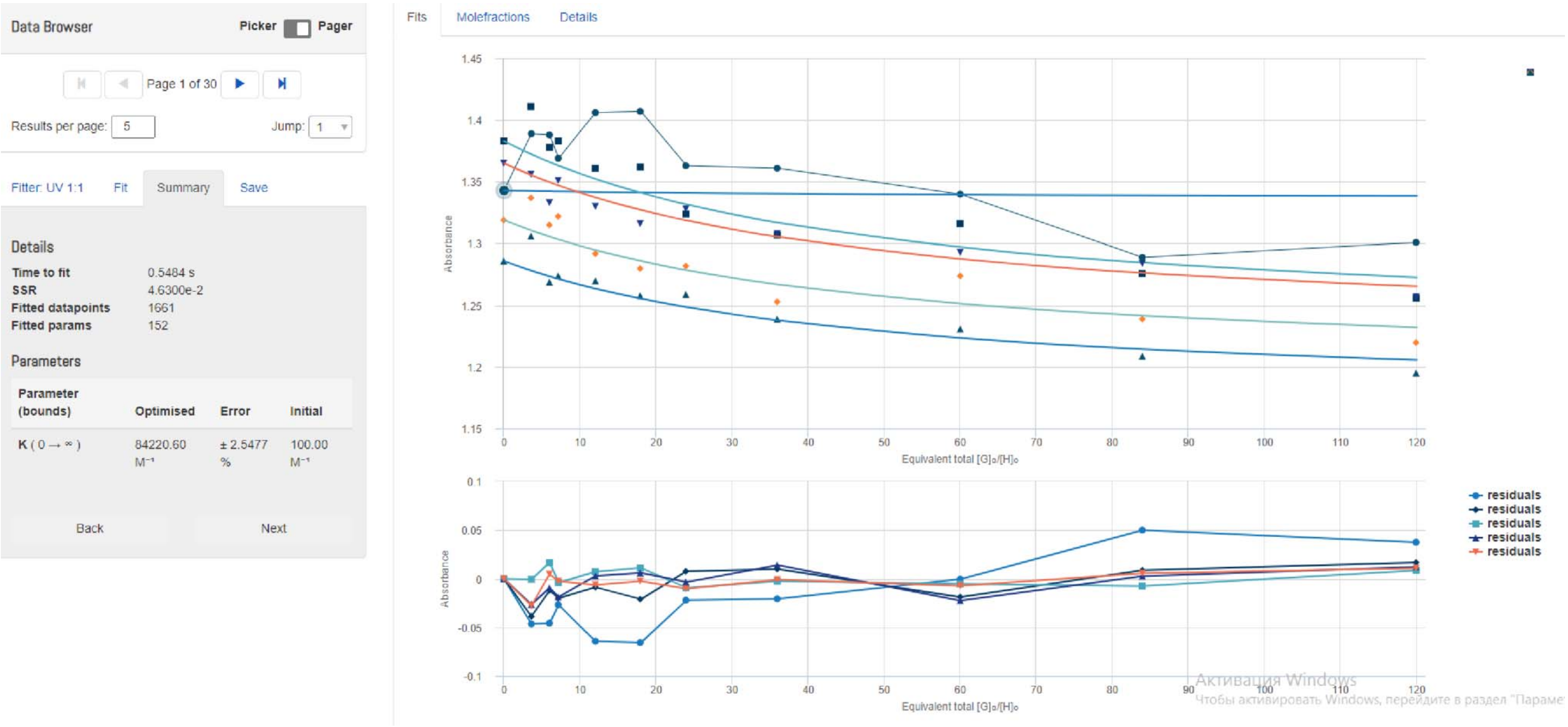

Fig. S24. Electron absorption spectra ($C_{macrocycle}$ = 3 × 10^{-5} M, C_{GIS} = 3 × 10^{-4} M) in Tris-HCl buffer: (a) pillar[5]arene 1/GIS; (b) pillar[5]arene 2/GIS; (c) pillar[5]arene 3/GIS; (d) pillar[5]arene 4/GIS.

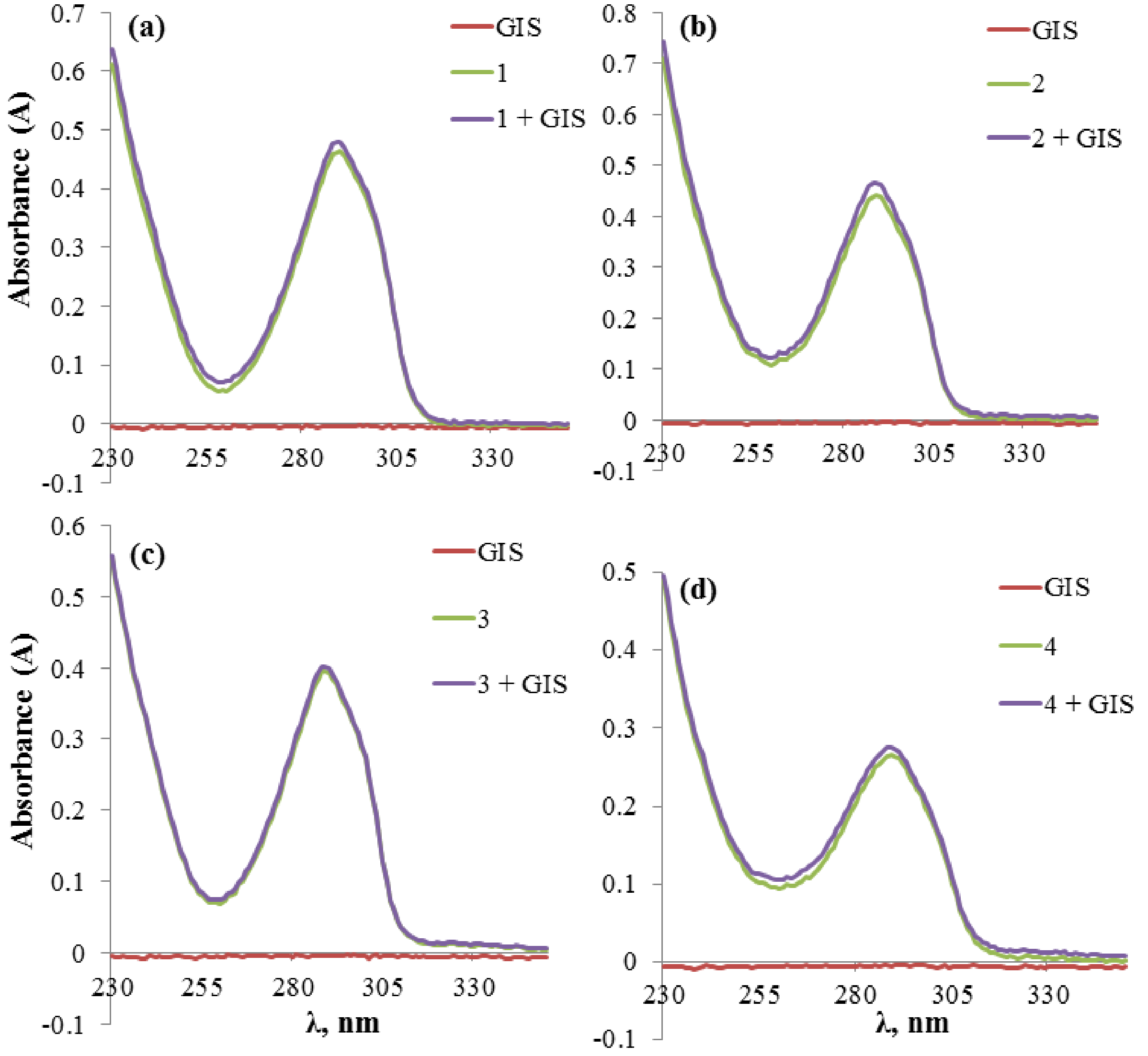

Fig. S25. (a) Electron absorption spectra of pillar[5]arene 1/DNA in Tris-HCl buffer ($C_{macrocycle}$ = 3 × 10^{-6} M, C_{DNA} = 2.5 × 10^{-7} M), pink arrow means hypochromic effect; (b) UV-Vis spectra of mixtures of DNA (2.5 × 10^{-7} M) with different concentrations of pillar[5]arene 1 (from 1 to 10-fold excess). Pink arrow means hypochromic effect with concentration increasing.

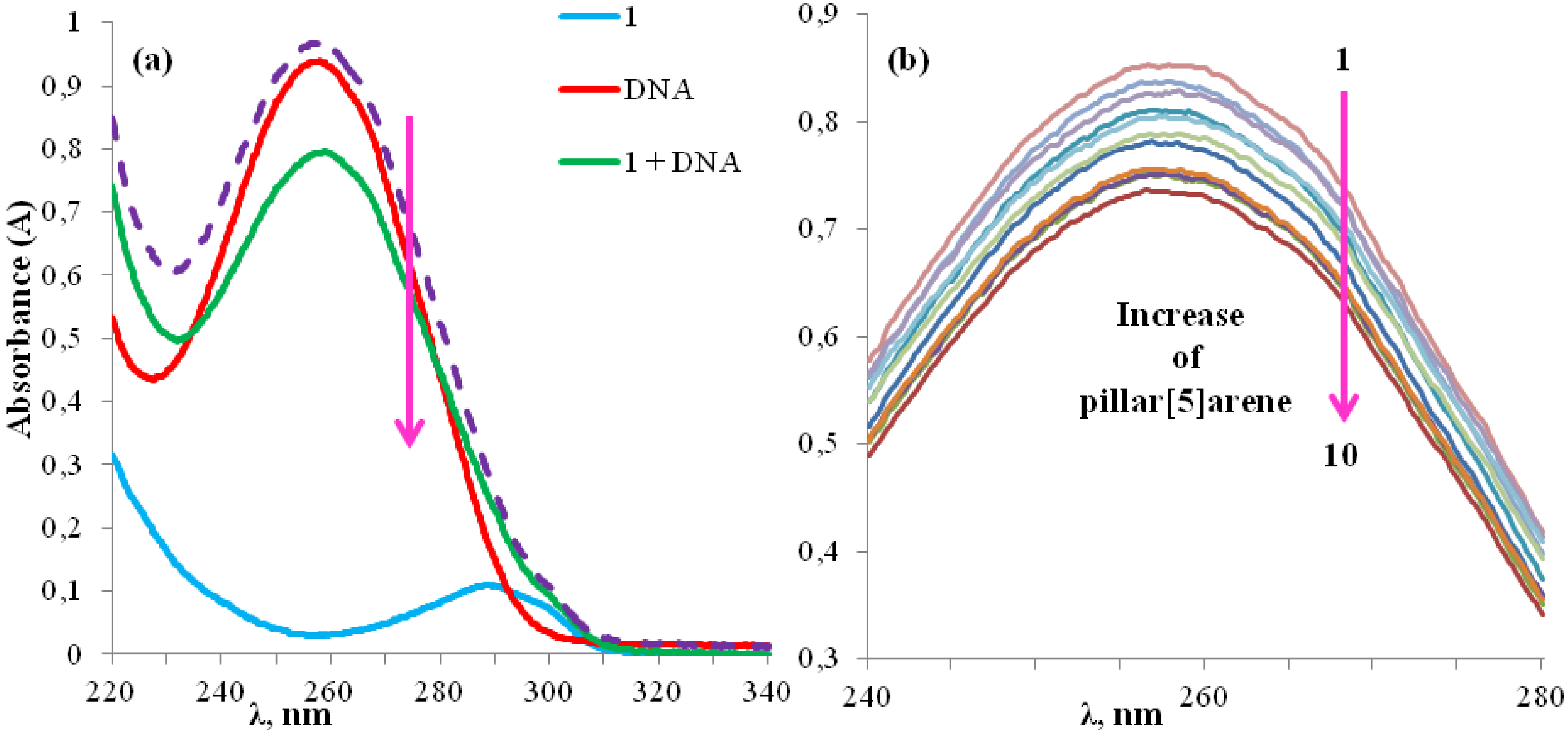

Fig. S26. Bindfit (Fit data to 1:1, 1:2 and 2:1 Host-Guest equilibria) Screenshots taken from the summary window of the website supramolecular.org. This screenshots shows the raw data for UV-vis titration of DNA with pillar[5]arene 4 in Tris-HCl buffer, the data fitted to 1:1 binding model.

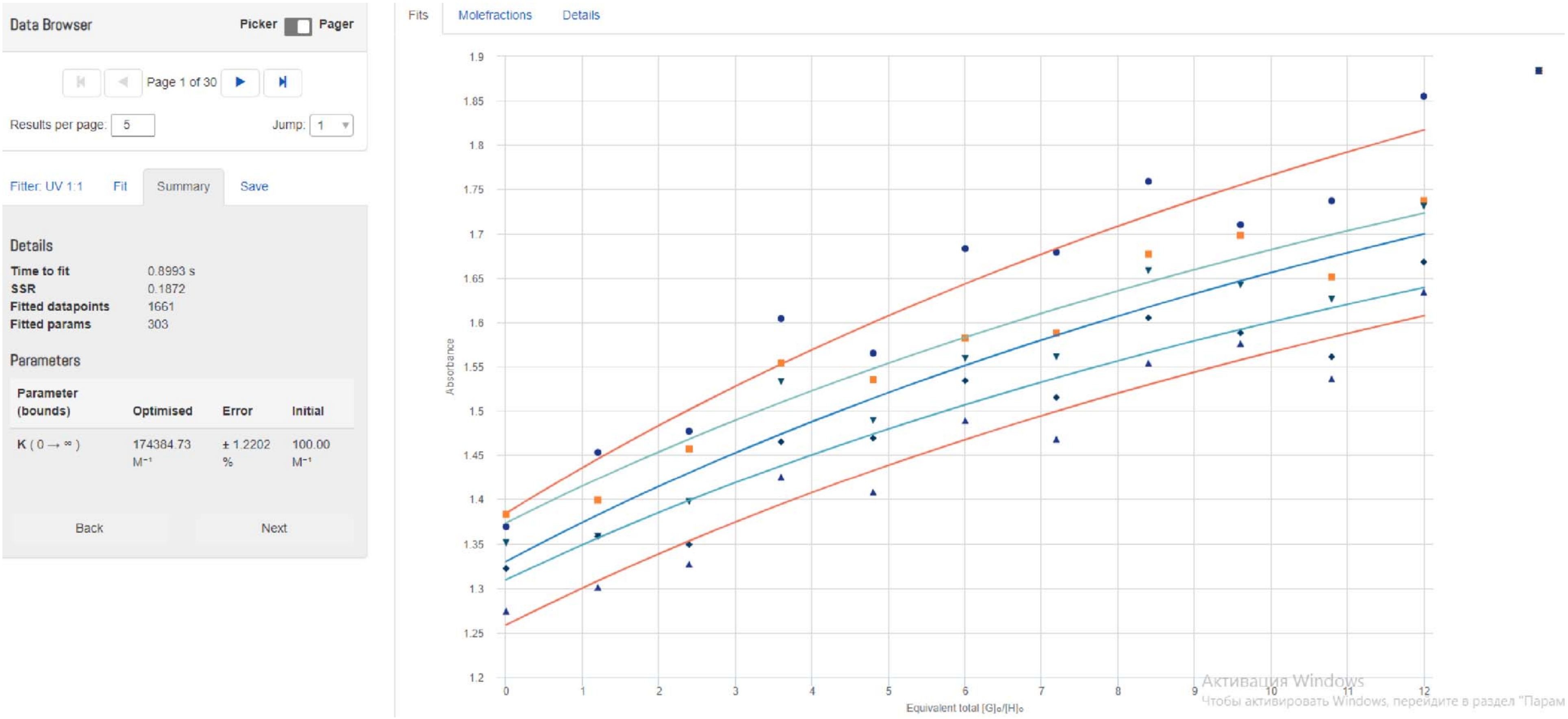

Fig. S27. Bindfit (Fit data to 1:1, 1:2 and 2:1 Host-Guest equilibria) Screenshots taken from the summary window of the website supramolecular.org. This screenshots shows the raw data for UV-vis titration of DNA with pillar[5]arene 1 in Tris-HCl buffer, the data fitted to 1:1 binding model.

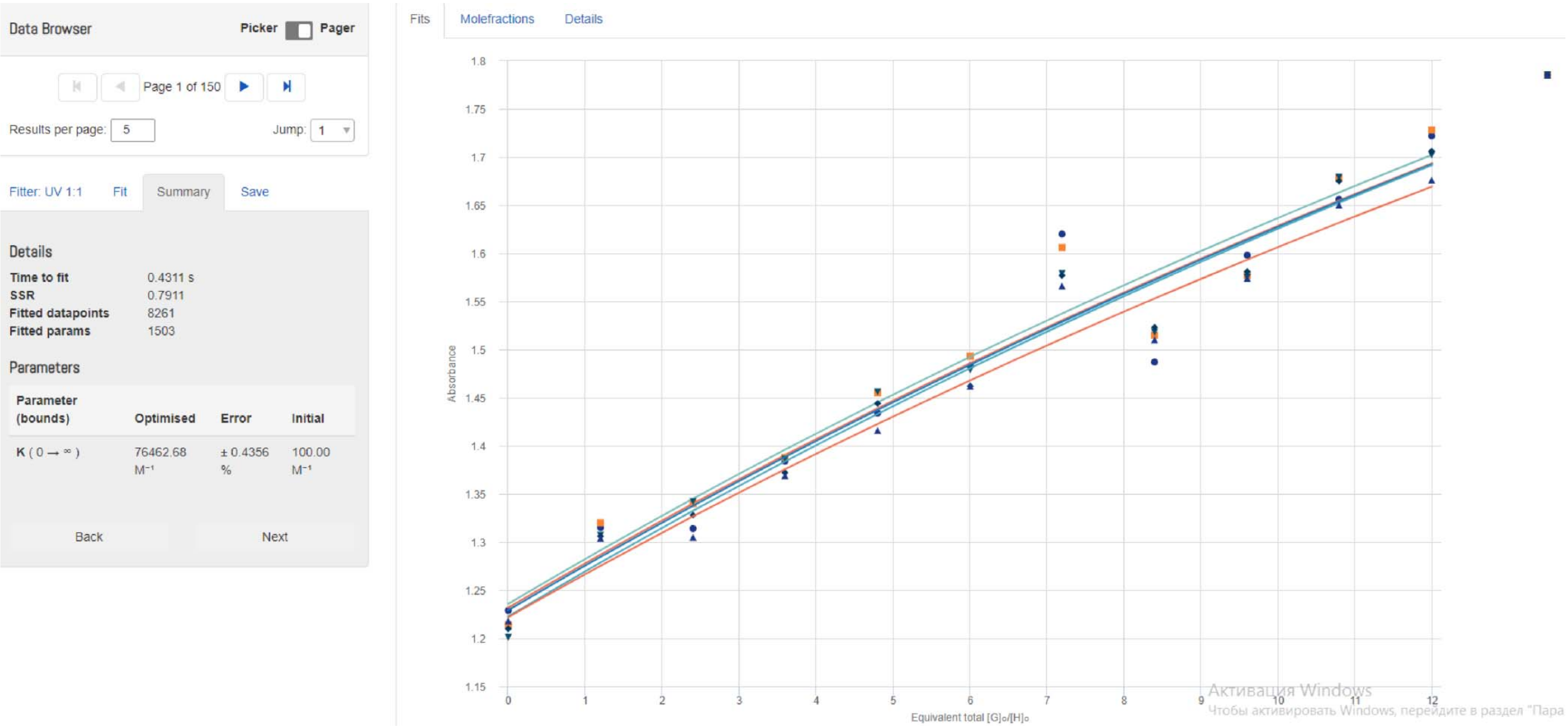

Fig. S28. Electron absorption spectra of (2 + DNA) with GIS (a) and (3 + DNA) with GIS (b) in Tris-HCl buffer ($C_{macrocycle}$ = 3 × 10^{-6} M, C_{DNA} = 2.5 × 10^{-7} M, C_{GIS} = 3 × 10^{-6} M), pink arrow means hyperchromic effect. UV-Vis spectra of mixtures of (2 + DNA) (c) and (3 + DNA) (d) with different concentrations of GIS (from 0.3 to 10-fold excess), pink arrow means hyperchromic effect with concentration increasing.

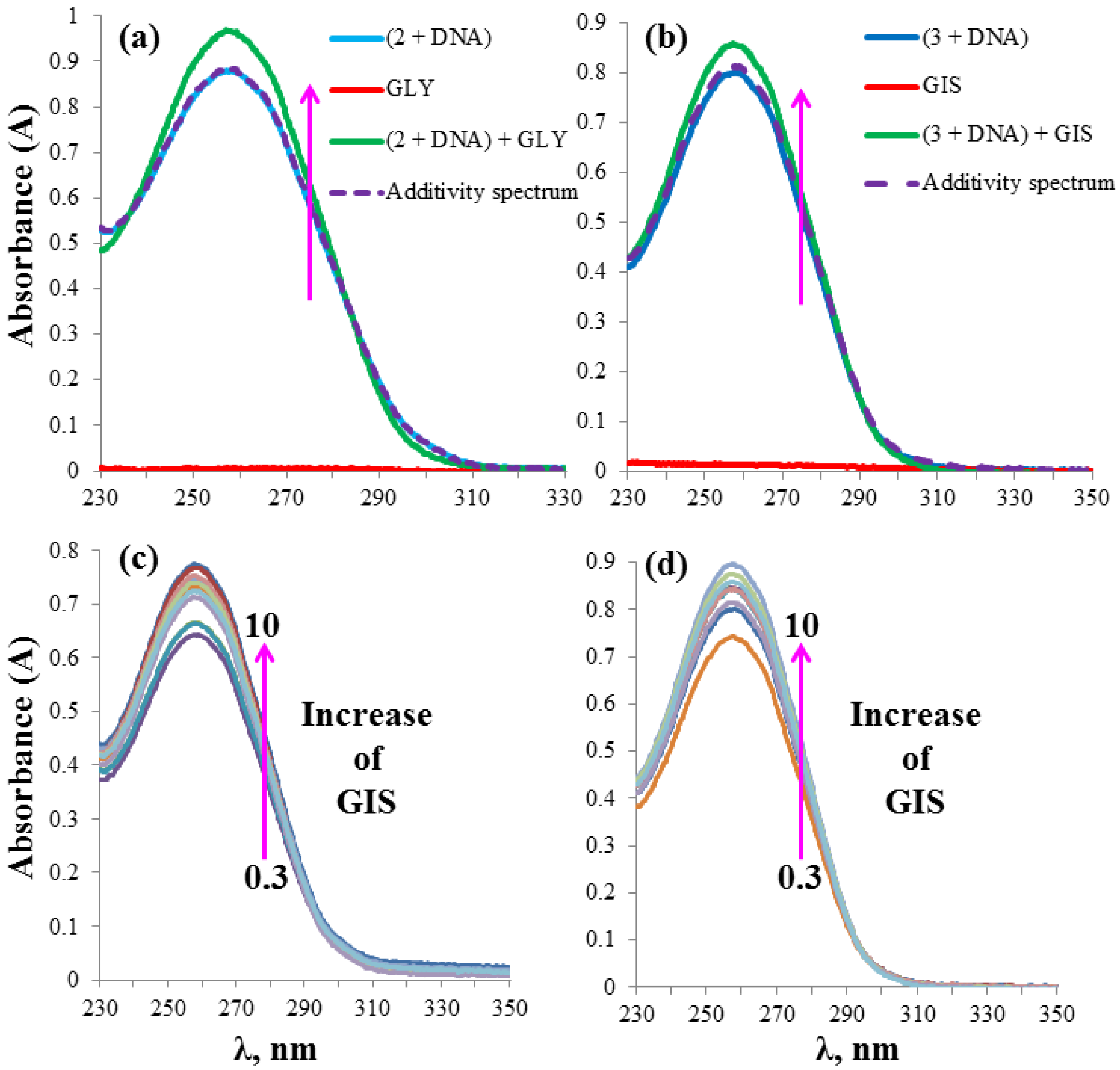

Fig. S29. Bindfit (Fit data to 1:1, 1:2 and 2:1 Host-Guest equilibria) Screenshots taken from the summary window of the website supramolecular.org. This screenshots shows the raw data for UV-vis titration of (1 + DNA) with GIS in Tris-HCl buffer, the data fitted to 1:1 binding model (a), 1:2 binding model (b) and 2:1 binding model (c).

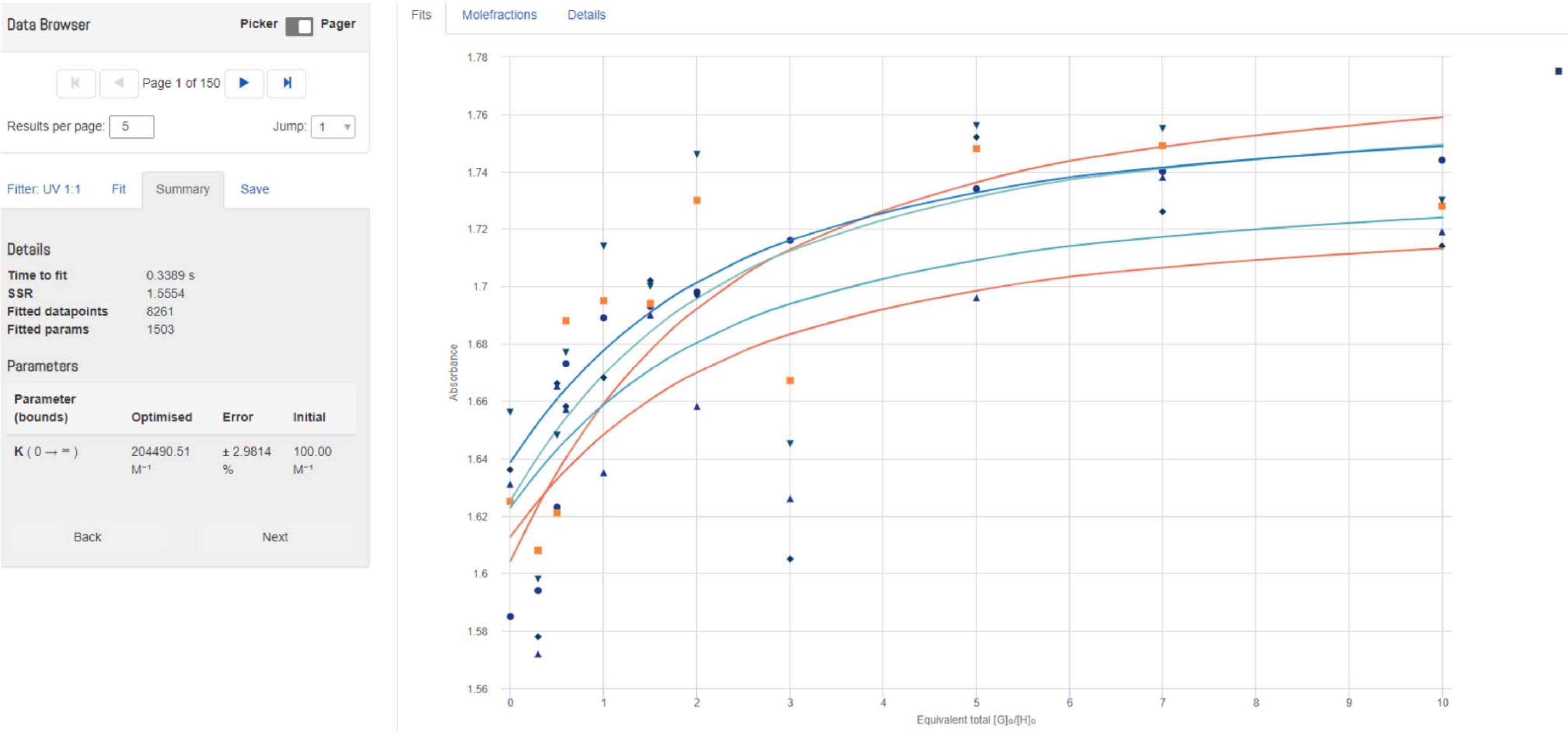

Fig. S30. Bindfit (Fit data to 1:1, 1:2 and 2:1 Host-Guest equilibria) Screenshots taken from the summary window of the website supramolecular.org. This screenshots shows the raw data for UV-vis titration of (2 + DNA) with GIS in Tris-HCl buffer, the data fitted to 1:1 binding model.

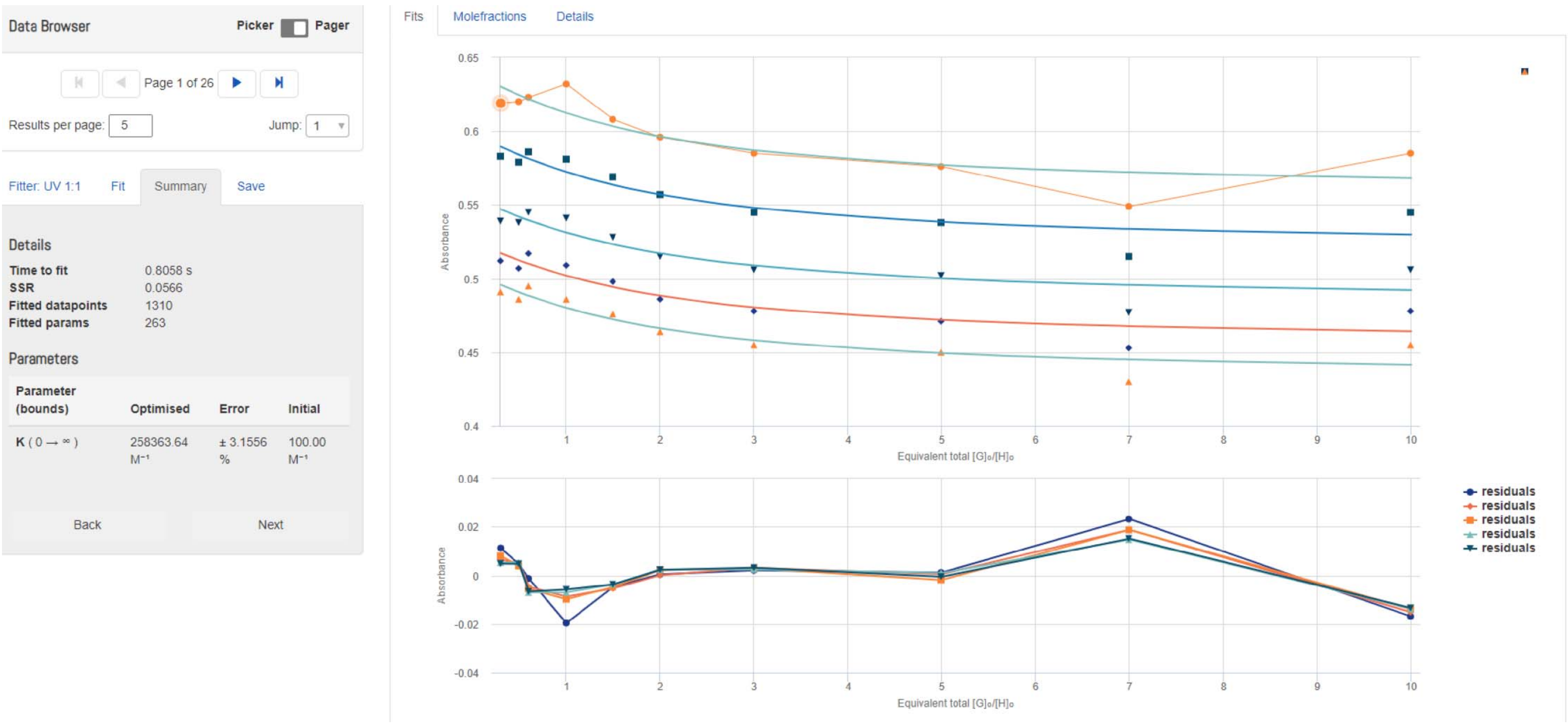

Fig. S31. Bindfit (Fit data to 1:1, 1:2 and 2:1 Host-Guest equilibria) Screenshots taken from the summary window of the website supramolecular.org. This screenshots shows the raw data for UV-vis titration of (3 + DNA) with GIS in Tris-HCl buffer, the data fitted to 1:1 binding model.

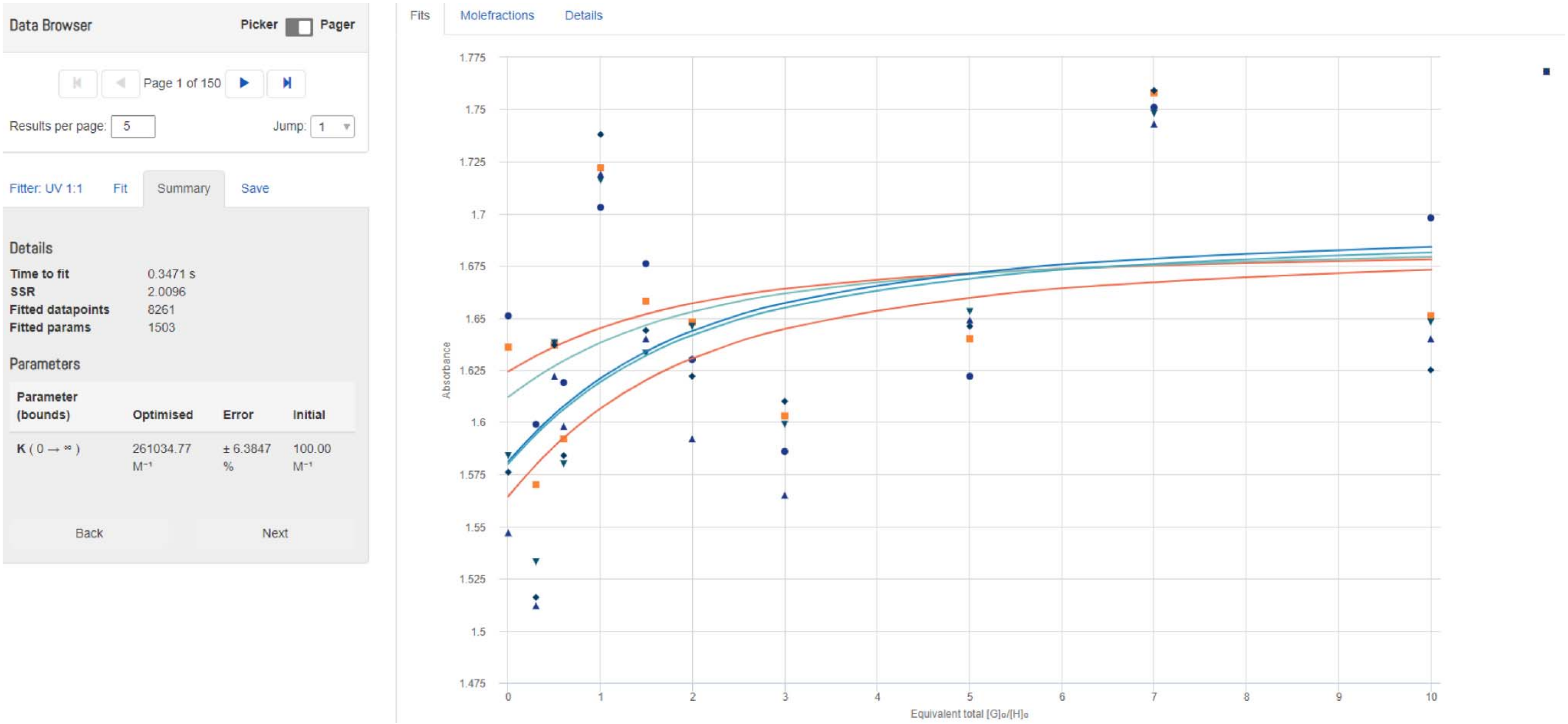

Fig. S32. Bindfit (Fit data to 1:1, 1:2 and 2:1 Host-Guest equilibria) Screenshots taken from the summary window of the website supramolecular.org. This screenshots shows the raw data for UV-vis titration of (4 + DNA) with GIS in Tris-HCl buffer, the data fitted to 1:1 binding model.

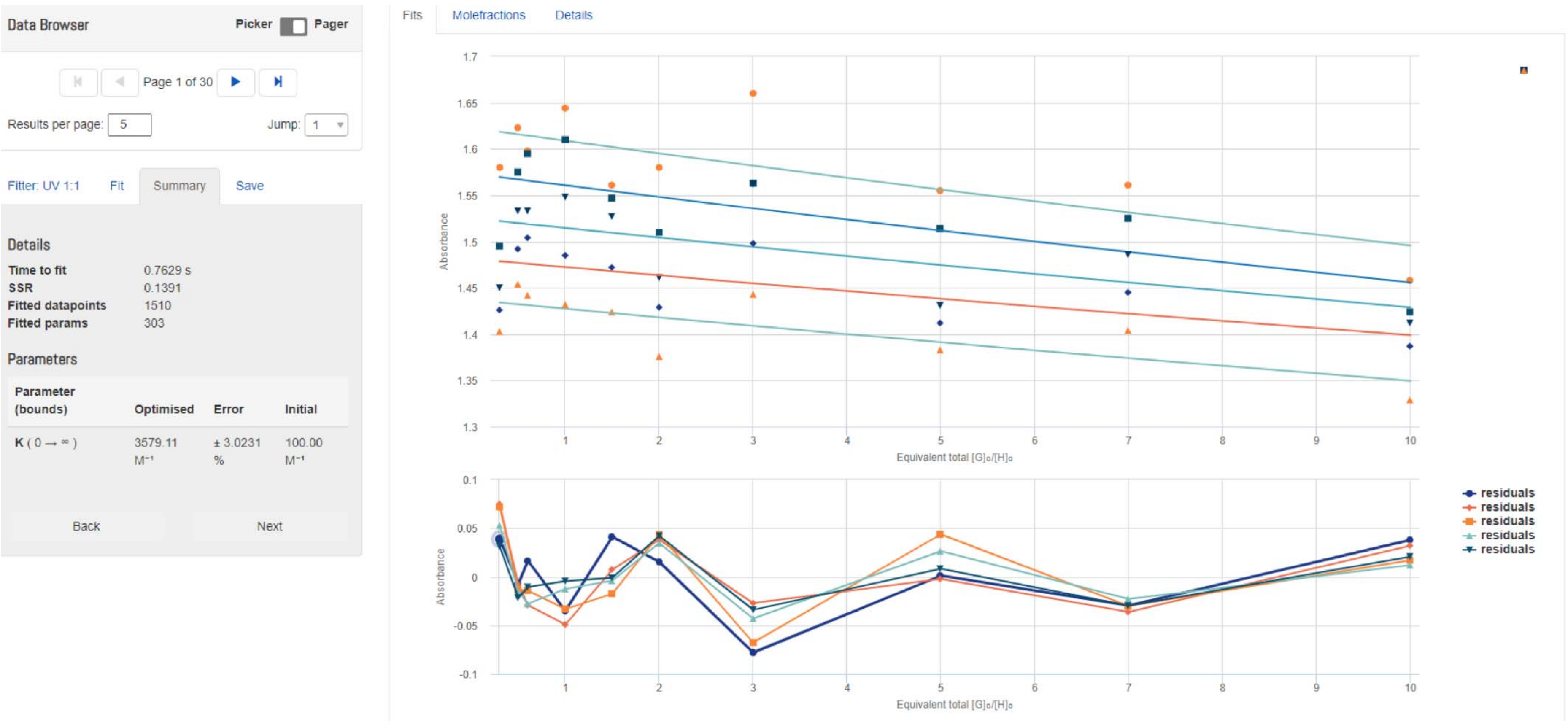

Fig. S33. (a) Size distribution of particles by intensity for associate (2 + DNA) + GIS (C_2=3×10^{-6} M, C_{DNA}=2.5×10^{-7} M, C_{GIS}=3×10^{-6} M) in Tris-HCl; (b) Zeta potential distribution for associate (2 + DNA) + GIS (C_2=3×10^{-6} M, C_{DNA}=2.5×10^{-7} M, C_{GIS}=3×10^{-6} M) in Tris-HCl.

(a)

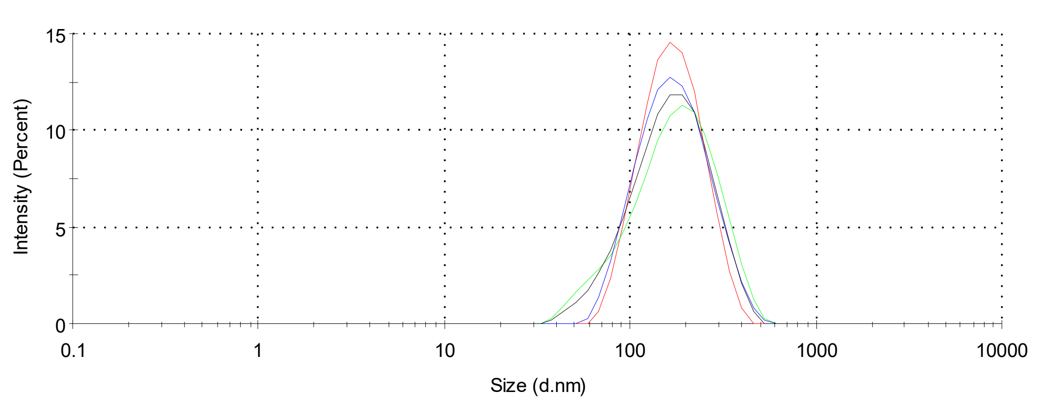

(b)

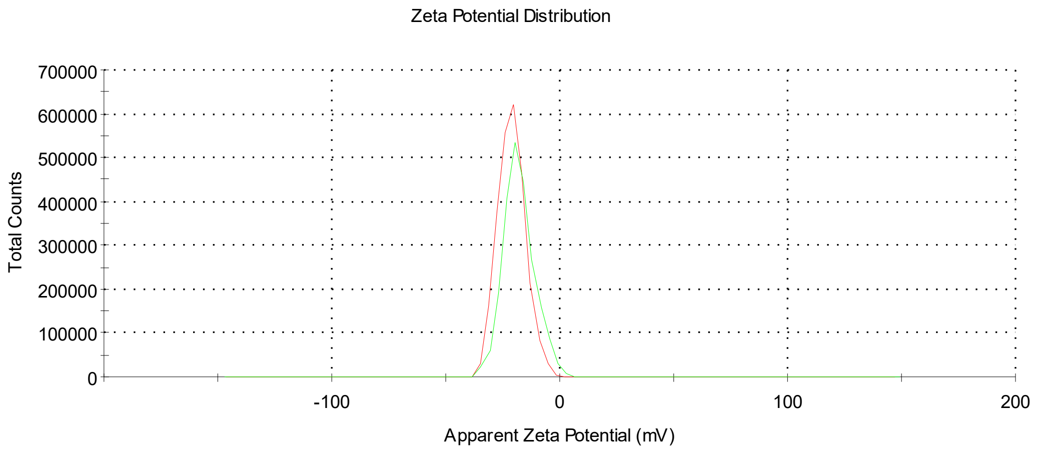

Fig. S34. Cytotoxicity of pillar[5]arenes 1, 3 and 4 on cell line *A549* and *LEC*.

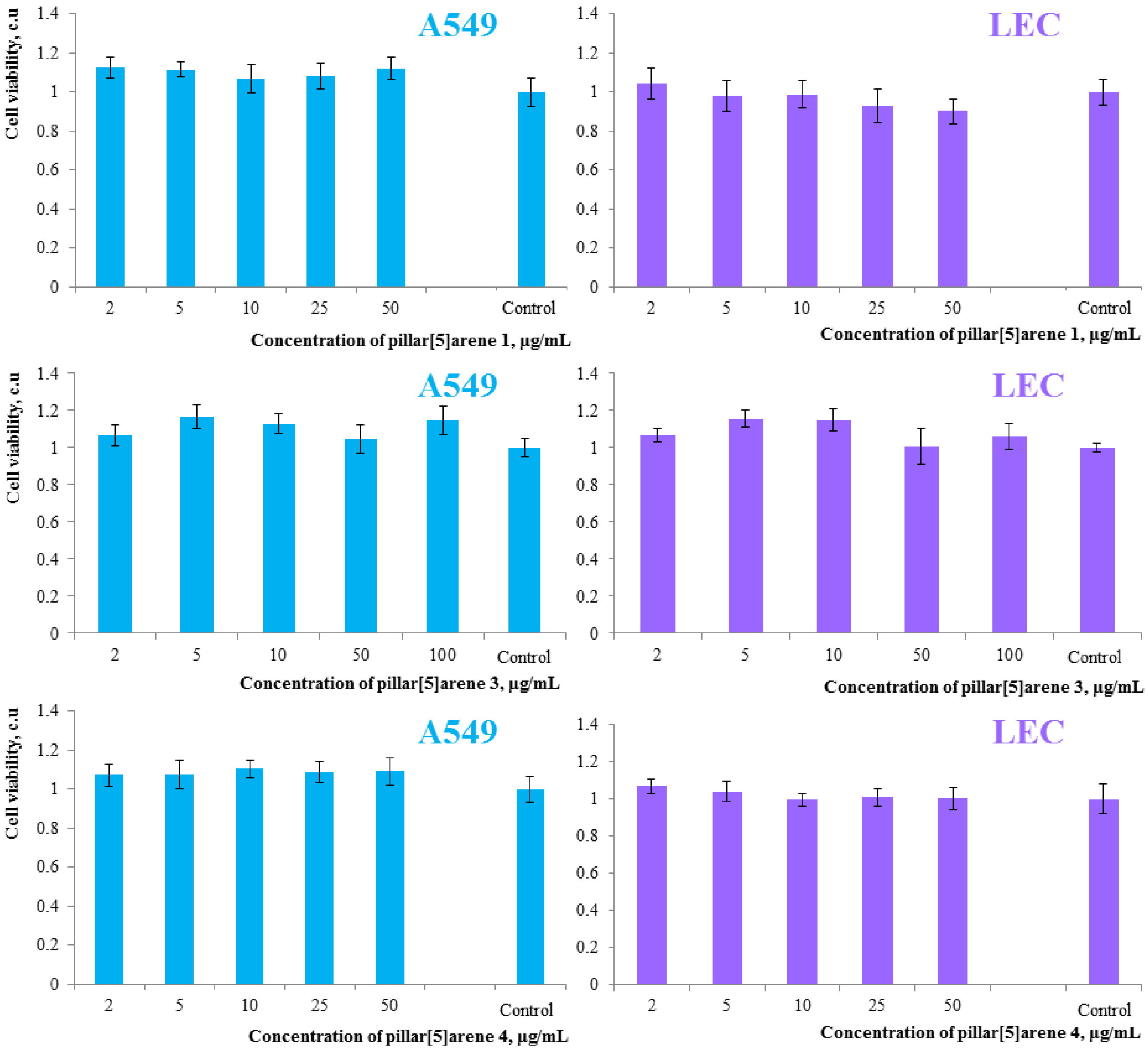

References

1. Nazarova, A.; Shurpik, D.; Padnya, P.; Mukhametzyanov, T.; Gragg, P.; Stoikov, I. Self-assembly of supramolecular architectures by the effect of amino acid residues of quaternary ammonium pillar[5]arenes. *Int. J. Mol. Sci*. **2020**, *21*, Art. Number 7206. doi 10.3390/ijms21197206
2. Mosmann, T. Rapid colorimetric assay for cellular growth and survival: Application to proliferation and cytotoxicity assays. *J. Immunol. Methods*. **1983**, *65*, 55–63. doi 10.1016/0022-1759(83)90303-4
3. Pozarowski, P.; Grabarek, J.; Darzynkiewicz, Z. Flow cytometry of apoptosis. *Curr. Protoc. Cytom*. **2003**. doi 10.1002/0471142956.cy0719s25
